# Evaluation of Multi-tRNA Synthetase Complex by Multiple Reaction Monitoring Mass Spectrometry Coupled with Size Exclusion Chromatography

**DOI:** 10.1371/journal.pone.0142253

**Published:** 2015-11-06

**Authors:** Seong-Jun Park, Hee-Sung Ahn, Jun Seok Kim, Cheolju Lee

**Affiliations:** 1 Center for Theragnosis, Biomedical Research Institute, Korea Institute of Science and Technology, Seoul 136–791, Republic of Korea; 2 Department of Biological Chemistry, University of Science and Technology, Daejeon 305–333, Republic of Korea; Moffitt Cancer Center, UNITED STATES

## Abstract

Eight aminoacyl-tRNA synthetases (M, K, Q, D, R, I, EP and LARS) and three auxiliary proteins (AIMP1, 2 and 3) are known to form a multi-tRNA synthetase complex (MSC) in mammalian cells. We combined size exclusion chromatography (SEC) with reversed-phase liquid chromatography multiple reaction monitoring mass spectrometry (RPLC-MRM-MS) to characterize MSC components and free ARS proteins in human embryonic kidney (HEK 293T) cells. Crude cell extract and affinity-purified proteins were fractionated by SEC in non-denaturing state and ARSs were monitored in each fraction by MRM-MS. The eleven MSC components appeared mostly in earlier SEC fractions demonstrating their participation in complex formation. TARSL2 and AIMP2-DX2, despite their low abundance, were co-purified with KARS and detected in the SEC fractions, where MSC appeared. Moreover, other large complex-forming ARS proteins, such as VARS and FARS, were detected in earlier fractions. The MRM-MS results were further confirmed by western blot analysis. Our study demonstrates usefulness of combined SEC-MRM analysis for the characterization of protein complexes and in understanding the behavior of minor isoforms or variant proteins.

## Introduction

Aminoacyl tRNA synthetases (ARSs) are key enzymes that catalyze the attachment of specific amino acid to their cognate tRNAs, which is the first step of protein synthesis [1]. Because the aminoacylation catalyzed by ARSs prevails in every living organism, ARSs are essential component for protein synthesis. To ensure translation process, ARSs encompass editing processes which hydrolyze misactivated amino acids or mischarged tRNAs [2]. In addition to their canonical functions in translation and editing, recent studies suggest that non-canonical functions of ARSs, which acquired additional domains or occurred alternative splicing, are associated with human diseases [3, 4]. Among twenty ARSs, eight ARSs (RARS, DARS, QARS, EPRS, LARS, IARS, KARS, and MARS) with three nonsynthetase components, aminoacyl tRNA synthetase complex-interaction multifunctional protein (AIMP) 1, 2 and 3, are known to form a supramolecular multi-tRNA synthetase complex (MSC), whose molecular weight has been proposed to be about 1.5 MDa [5, 6]. The MSC is regarded to increase the efficiency of protein synthesis through channeling processes for tRNA and act as a reservoir to control non-canonical functions of ARSs [7, 8].

ARSs are not only involved in protein synthesis, but also in the regulation of various signaling pathways [3]. ARSs contain unique extensions and domains, which endow them with functional diversity through the interactions with various cellular partners. Our previous result obtained through affinity purification mass spectrometry (AP-MS) suggests that threonyl-tRNA synthetase like protein 2 (TARSL2) interacts with MSC and variants such as AIMP2-DX2, an exon 2-deleted splicing variant of AIMP2, and AIMP1 isoform 2, which has 24 additional amino acids at the N-terminus of AIMP1, interact with lysyl-tRNA synthetase (KARS) [9]. Although tandem affinity purification and x-crystallography has demonstrated similarity in composition of complexes from *Caenorhabditis elegans* and mammalian system, accurate composition of MSC components remain unclear because the interaction network of ARSs is very complex and the native multi-protein complex is unstable to proteolysis [10–12].

Protein complexes have been purified using many biochemical techniques including ion exchange chromatography and size-exclusion chromatography (SEC) before MS analysis of proteins [13, 14]. SEC has been used as an internal step in classical protein purification process and less commonly combined with MS based proteomic analysis for protein complex. However, SEC provides a unique advantage over other chromatographic methods because elution time of protein complex changes according to its molecular weight. Thus, SEC coupled to MS has become the strategy of choice for analysis of intact protein complexes in plants and mammalian cells [15, 16]. Recently, MS-based quantification, especially multiple reaction monitoring mass spectrometry (MRM-MS), has been utilized for the accurate quantification of multiple target proteins without employing antibodies [17–19]. Compared with conventional mass spectrometric technique, MRM-MS enables multiple low abundance targets to be simultaneously detected and quantified [18, 20]. In addition, MRM-MS is a rapid and selective biological analysis strategy. One or more surrogate peptides that are unique to a target protein are used to quantify the target because of their high selectivity and specificity for targets. Due to high selectivity, MRM-MS can differentiate even a single amino acid variation, which is intractable by antibody-based immunoassay.

In this study, we combined SEC with MRM-MS to characterize MSC components and free ARS proteins in HEK 293T cell line. We separated intact MSC from free ARSs by SEC and quantified the amount of ARS proteins in each SEC fraction by MRM-MS. Herein, we demonstrate the utility of this strategy for identification of intact protein complex and its application in detecting and quantifying protein isoforms and variant proteins.

## Materials and Methods

### Transfection and sample preparation

The HEK 293T cells were maintained in Dulbecco’s Modified Eagle Medium (Gibco, Rockville, MD, USA) supplemented with 10% fetal bovine serum (Gibco) and 1% penicillin/streptomycin (Gibco) in a humidified incubator in an atmosphere of 95% air and 5% CO_2_ at 37°C. Cells were grown to ~80% confluence at the initiation of the experiment. KARS was cloned into a vector, pIRES2-EGFP-SBP, engineered to express fusion proteins with N-terminal S, FLAG and streptavidin binding peptide (SBP) tags. The construct was transiently transfected into HEK 293T cell line using X-tremeGENE HP DNA Transfection Reagent (Roche Diagnostics, Indianapolis, IN, USA) for 36 h at 37°C. The HEK 293T and transfected cells (KARS^oe^) were harvested at a confluence of 80~90% and lysed using NETN buffer containing 20 mM Tris-HCl, pH 7.5, 1 mM EDTA, 150 mM NaCl, 0.1% Nonidet P-40, and Protease Inhibitor Cocktail (Roche Diagnostics). Cell lysate was pelleted by centrifugation (12,000 rpm, 10 min, 4°C) and the supernatant was collected. Subsequently, protein amount was measured by BCA protein assay (Thermo Scientific, Rockford, IL, USA) and KARS expression was confirmed by western blot.

### Affinity purification

Streptavidin affinity purification was performed as described previously [9]. Briefly, 50 μL of streptavidin agarose beads (Thermo Scientific) were activated and equilibrated with 500 μL of NETN buffer twice. KARS^oe^ cell lysate (2 mg) was added to the agarose beads and subjected to rotation (10 rpm, 2 h, 4°C). After incubation, the mixtures were washed three times with NETN buffer. The bound proteins were eluted employing 30 μL of NETN buffer containing biotin (0.82 mM) using a 0.22 μm centrifugal filter device (Millipore, Billerica, MA, USA). The elution step was repeated twice.

### Size exclusion chromatography

The volume of supernatants from HEK 293T (3 mg protein), KARS^oe^ (3 mg protein) and streptavidin affinity purification from KARS^oe^ cells (denoted as KARS^oe^-AP afterwards) (200 μg protein) was adjusted to 500 μL using NETN lysis buffer. These samples were loaded onto a Superdex 200 10/300 GL column in an ÄKTA FPLC system (GE healthcare, Uppsala, Sweden), and eluted with PBS buffer at an optimal flow rate of 0.5 mL/min. The eluate was monitored by UV absorption at 280 nm, collected in 500 μl fractions, and exchanged with 50 mM Tris-Cl (pH 7.5) plus 6 M urea employing 3K Amicon ultra centrifugal filter (Millipore). The amount of protein in each fraction was measured by BCA protein assay.

### In-solution digestion

Equal volume of buffer-exchanged fractions (40 μL) was reduced with 5 mM dithiothreitol (DTT) at 25°C for 1 h and alkylated with 15 mM iodoacetamide (IAA) at 25°C for 1 h in the dark. The samples were diluted 10-fold with 50 mM Tris-Cl (pH 7.5) to decrease the concentration of urea in the sample to less than 1 M. For tryptic digestion, sequencing-grade modified trypsin (Promega, Madison, WI, USA) was added to the sample with an enzyme to protein ratio of 1/50 (w/w) and incubated at 37°C for 16 h. To stop the reaction, 0.3% trifluoroacetic acid (TFA) (pH < 3.0) was added. Stable isotope standard (SIS) peptides were spiked into the digests. In the fractions of HEK 239T and KARS^oe^, 4 pmol of SIS peptides were added, while 2 pmol of SIS peptides were added to all fractions of KARS^oe^-AP eluate. The digests were then desalted with a C-18 spin column cartridge (Nest group, Southborough, MA, USA), and the eluates were dried in a vacuum centrifuge (miVac Duo Concentrator, Genevac, Suffolk, UK) and stored at -20°C until use.

### Synthetic ARSs peptide

We selected 38 surrogate peptides representing target ARS proteins from the Uniprot database (released as of 2014.02) using the BLAST search tool. Peptide selection was based on the criteria, we had presented in our previous paper [18]. Briefly, peptides containing of 6–20 amino acids were chosen. Peptides containing amino acids prone to chemical modification, such as methionine and N-terminal glutamine were avoided. M/z 1250 is the maximum scan range of the instrument; hence, peptides with m/z values less than 1250 Da were selected. Doubly or triply charged precursor ions were chosen since these ions were reproducibly detected and stable for quantification. SIS peptides incorporating C-terminal [^13^C_6_, ^15^N_2_] lysine and [^13^C_6_, ^15^N_4_] arginine were purchased from 21st Century Biochemicals (Marlboro, MA, USA). For each peptide, amino acid analysis (AAA) was executed and the absolute amount of peptide was provided by the vendor. The peptides were re-solubilized in 200 μL of 20% acetonitrile and 0.1% formic acid and stored at -80°C until use.

### LC-MRM-MS setup and optimization

Multiple Reaction Monitoring (MRM) quantitation was performed on QTrap5500 equipped with a nanoelectrospray ion source (ABSciex, Foster City, CA, USA). The MS was operated in positive mode with the following parameters: ion spray voltage of 2100 V, curtain gas at 20 psi, nebulizer gas at 25 psi, resolution at 0.7 Da (unit resolution) for Q1 and Q3, interface temperature at 150°C, and scan mass range of 300−1250 m/z. The collisional energy (CE), collisional cell exit potential (CXP) and declustering potential (DP) were optimized by direct infusion using Turbospray. We monitored three most intense y-ions for SIS peptides during MRM measurements except for the AIMP2-DX2 peptide, for which top 5 y-ions were monitored (S1 Table). The identical experimental conditions were used for endogenous peptides. Quantification experiments were performed using a scheduled LC-MRM mode with MRM detection window of 600 s and cycle time of 1.5 s. For chromatographic separation, an Eksigent nanoLC-Ultra 2D plus (Eksigent Technologies, CA, USA) interfaced with NanoFlex system (Eksigent Technologies, Redwood, CA, USA) was used. Samples were reconstituted with 20 μL of 2% acetonitrile and 0.1% formic acid, injected with a full sample loop injection of 1 μL, and separated in Nano cHiPLC ReproSil-Pur C18- columns (75 μm i.d, 15 cm length, pore size 120 Å, particle size 3 μm; Eksigent Technologies). The column was priory equilibrated with 95% mobile phase A (0.1% formic acid in water) and 5% mobile phase B (0.1% formic acid in acetonitrile). Peptides were eluted with a gradient of 5–10% mobile phase B for 4 min, 10–25% for 30 min, 25–60% for 3 min, 60%-60% for 3 min, 60–5% for 1 min, 5%-5% for 9 min at a flow rate 300 nL/min.

### Analysis of MRM-MS data

Raw files of MRM-MS data (*.wiff) from Analyst software (Version 1.5.2, ABSciex) were processed in Skyline (version 2.6.0) [21]. The program was designed to extract quantitative information from MRM measurements based on extracted ion chromatogram (XIC). The most intense transition of each peptide was selected for the quantification and the other transitions together with the most intense transition were used for peak assignment and validation. The identification and quantification of endogenous peptides were based on the corresponding SIS peptides. Finally, 25 ARS proteins were quantified by a single representative peptide having the greater quantitative values because that peptide might have been generated with the higher digestion efficiency (S2, S3 and S4 Tables). All statistical data were analyzed by using R package (version 3.0.3) and Excel 2010 (version 14.0, Microsoft Office). Duplicate MRM measurements were performed for all SEC fractions. Coefficient of variation (CV) in measurement for each ARS protein was calculated as follows:

$$\mathrm{CV}=\sqrt{\sum_{k}^{N}\frac{{\left(x_{k1}-x_{k2}\right)}^{2}}{2N}}/\sum_{k}^{N}\frac{\left(x_{k1}+x_{k2}\right)}{2N}$$

where *x*_k1_ and *x*_k2_ are the MRM signal intensities of duplicated measurements at *k*-th SEC fraction and N is the total number of fractions [22].

### Western blot analysis

Equal volume of buffer exchanged SEC fraction sample (5 μL) was separated by SDS-PAGE and then transferred to a PVDF membrane (GE Healthcare) using a Bio-Rad Trans-blot Cell system (Bio-Rad, Hercules, CA, USA). Electrophoretic transfer to the PVDF membrane was performed at 300 mA for 2 hr. All membranes were blocked using 5% skim milk in TBS-T buffer (25 mM Tris, 190 mM NaCl and 0.05% Tween 20, pH 7.5) for 1 h at 25°C and incubated with primary antibody at 4°C for overnight. The membrane was washed three times with TBS-T and then incubated with IgG-HRP secondary antibodies at a dilution of 1:2000 for 1 h at RT. After washing, immunoreactive proteins were detected using Calarity^TM^ Western ECL Substrate (Bio-Rad). The primary antibodies used in this study were directed against the following proteins: FLAG (F3165, Sigma-Aldrich); AARS (ab155275), EPRS (ab31531), FARSA (ab54653), KARS (ab129080), LARS (ab31534), MARS (ab50793), RARS (ab31537), TARS (ab58240), TARSL2 (ab93186), AIMP1 (ab96506), AIMP2 (ab101840) and YARS (ab50961) (Abcam, Cambridge, UK).

## Results and Discussion

**Fig 1:**
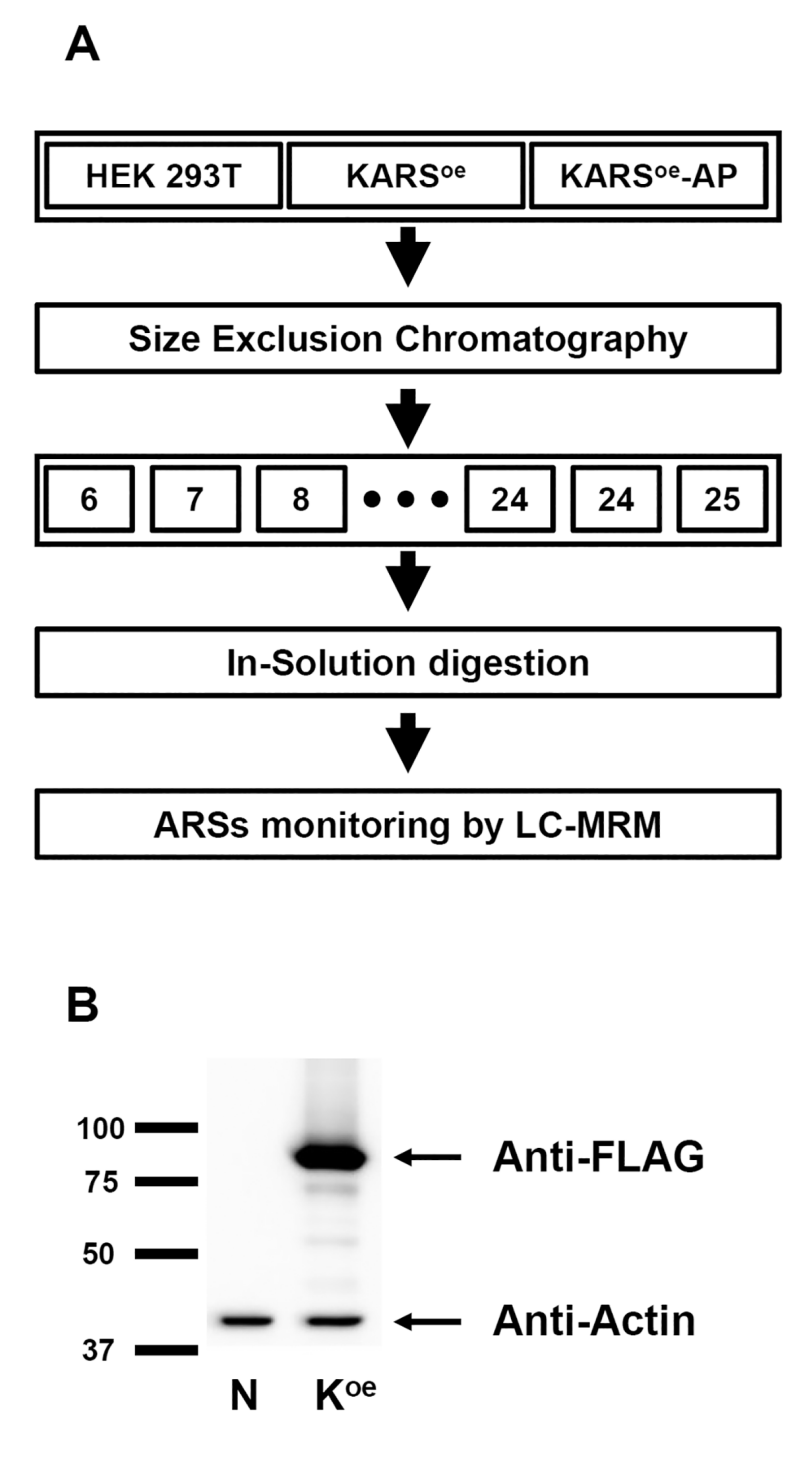
Overall workflow for detection of ARS proteins. **(A)** Overall workflow. Two cell lysates (HEK 293T and KARS^oe^) and affinity purification eluate (KARS^oe^-AP) were fractionated using SEC. Each fraction was digested with trypsin and spiked with the SIS peptide. After C18 clean-up, the digest was analyzed by LC-MRM-MS. All the data were analyzed by Skyline. **(B)** Overexpression of KARS tagged with S/FLAG/SBP in HEK 293T cell was detected by western blot using anti-FLAG antibody. Actin was used as a loading control. N, HEK 293T cell; K^oe^, KARS-overexpressing cell.

**Fig 2:**
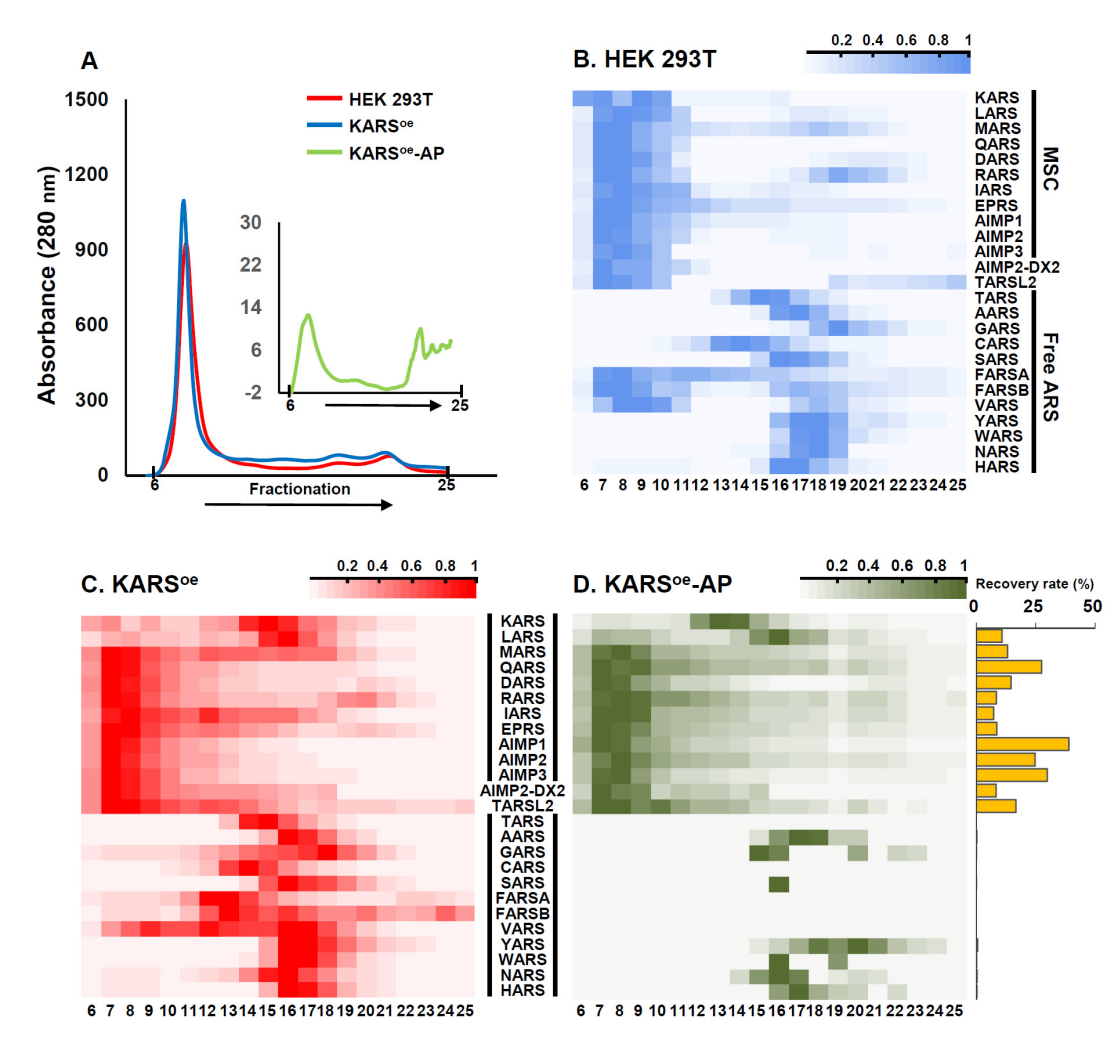
Two-dimensional MRM profile of ARS proteins in the fractions of size exclusion chromatography. **(A)** Two cell lysates and affinity purification eluate were injected on a Superdex 200 column and the elution profile was recorded by following the 280 nm absorbance. Red: HEK 293T cell lysate; Blue: KARS^oe^ cell lysate; Green; KARS^oe^-AP eluate. **(B, C and D)** The average amount of 25 ARS proteins in 20 fractions of HEK 293T (B), KARS^oe^ (C) and KARS^oe^-AP (D) from duplicated LC-MRM runs is represented as heat maps. The average value is normalized against the largest value among the 20 fractions (S2, S3 and S4 Tables). In the heat maps, each row represents an ARS protein; each column represents an SEC fraction. **(D, right panel)** The histogram represents recovery rate (%) of each ARS protein after affinity purification. The recovery rate is a relative value to that of KARS which was used as a bait.

Composition of intact MSC component is still uncertain due to high sensitivity caused by uncontrolled proteolysis and various transient interactions of ARS proteins. Additionally, a little clue about MSC composition concerning the assembly of its components has been obtained by reconstitution of sub-complex from recombinant proteins and by affinity purification [10, 23, 24]. In this study, we have established a new workflow to detect and quantify MSC components and free ARSs, which are not included in MSC. We used both HEK 293T and lysyl-tRNA synthetase (KARS)-overexpressing (KARS^oe^) cells. KARS^oe^ cells had also been used in our previous work (Fig 1B), where we identified TARSL2 and AIMP2-DX2 in KARS precipitate [9]. Cell lysates prepared from the two cell lines were fractionated by SEC into 20 consecutive fractions of equal volume. Subsequently, each fraction was analyzed by MRM-MS after digestion with trypsin. In a parallel experiment, we purified MSC by streptavidin affinity chromatography from KARS^oe^ cells (KARS^oe^-AP) before SEC (Fig 1). In summary, HEK 293T (3 mg), KARS^oe^ (3 mg) cell lysate and KARS^oe^-AP eluate (200 μg) were subjected to SEC (Fig 2A). SEC is a well-established technique used to separate proteins and protein complexes in solution based on their size and shape. Therefore, complex-forming protein will elute in the earlier fraction than the protein when it does not form a complex.

In order to monitor ARSs by MRM-MS, a unique peptide for each target protein should be selected and MRM parameters for the surrogate peptides need to be optimized. We followed the selection criteria for the MRM-assayable surrogate peptides, which had been well documented in our previous paper [18]. Peptide uniqueness of target proteins was confirmed by searching the amino acid sequence against the Uniprot protein sequence database as of 2014.02. The MRM peptides and their optimized MRM parameters are listed in Table 1. The peptide for AIMP2-DX2, SYGPAPGAGHVQDYGALK, spans exon 1/ exon 3 boundary, and thus differentiates the DX2 isoform from the wild-type canonical form. However, the peptide used for AIMP2 monitoring, SCENLAPFNTALK, represents all isoforms. A peptide unique to wild-type canonical form, SYGPAPGAGHVQEESNLSLQALESR, showed poor ionization efficiency, and was not observable within detection limits, which forced us to exclude it from further analysis. SIS peptides were spiked into each fraction, which served as internal standard to authenticate MRM signal by matching elution times and MRM transitions between SIS and endogenous peptides. All MRM peptides eluted at exactly the same retention time as their corresponding SIS peptides (S1 Fig). We found CV values of MRM measurement for all 25 ARSs less than 25% and with a median value of 4.1% (HEK 293T), 4.6% (KARS^oe^) and 16.2% (KARS^oe^-AP) (S2, S3 and S4 Tables). As a check for the reproducibility of SEC-MRM, we analyzed Pearson correlation coefficient (PCC) of MRM results between each pair of three biological replicates of the three different experimental conditions (HEK 293T, KARS^oe^ and KARS^oe^-AP). The median PCC of quantified ARS proteins was found as 0.81 for HEK 293T, 0.85 for KARS^oe^ and 0.88 for KARS^oe^-AP (S2 Fig). These results indicate that most ARS proteins were consistently separated and detected by SEC-MRM.

**Table 1:** MRM conditions for quantified proteotypic peptides representing 25 ARS proteins.

Gene name	Peptide sequence	light^a^		heavy^b^		charge state		ion	DP^c^	CE^d^	CXP^e^	RT^f^
		Q1	Q3	Q1	Q3	Q1	Q3					
AARS	GLEVTDDSPK	530.761	662.299	534.769	670.313	2	1	y6	41	21	14	15.16
AIMP1	GVPFEVK	388.221	310.176	392.228	314.183	2	2	y5	61	13	16	20.95
AIMP2	SC*ENLAPFNTALK	732.861	790.446	736.868	798.46	2	1	y7	116	29	36	28.68
AIMP2 DX2	SYGPAPGAGHVQDYGALK	596.629	769.392	599.3	773.399	3	2	y16	71	21	8	20.21
AIMP3	AIVQQWLEYR	653.351	894.447	658.355	904.455	2	1	y6	121	29	40	35.19
CARS	APVDITGQFEK	602.814	937.463	606.821	945.477	2	1	y8	141	27	18	24.56
DARS	VFSIGPVFR	511.295	775.446	516.299	785.454	2	1	y7	71	21	36	35.57
EPRS	NSEPAGLETPEAK	671.828	1012.531	675.835	1020.545	2	1	y10	86	27	44	15.95
FARSA	HWELTAEGEEIAR	514.251	674.347	517.587	684.355	3	1	y6	61	17	28	25.07
FARSB	NPGFEIIHGLLDR	494.268	456.254	497.605	459.59	3	3	y12	86	21	24	39.89
GARS	NNIIQTWR	522.783	703.389	527.787	713.397	2	1	y5	76	21	14	24.35
HARS	DQGGELLSLR	544.291	844.489	549.295	854.497	2	1	y8	51	25	8	27.71
IARS	ESVDHLTIPSR	418.554	359.204	421.89	369.212	3	1	y3	96	17	18	20.11
KARS	LIFYDLR	470.269	713.362	475.273	723.37	2	1	y5	86	19	14	33.9
LARS	VFASELNAGIIK	631.361	1015.578	635.368	1023.592	2	1	y10	81	25	44	30.72
MARS	TLPGSDWTPNAQFITR	902.455	795.389	907.459	800.393	2	2	y14	166	35	34	34.99
NARS	IGALEGYR	439.74	765.389	444.744	775.397	2	1	y7	106	21	34	18.55
QARS	LGYFSVDPDSHQGK	517.248	768.363	519.919	776.378	3	1	y7	66	25	34	23.4
RARS	LNDYIFSFDK	631.309	643.309	635.316	651.323	2	1	y5	91	27	6	36.87
SARS	YLIATSEQPIAALHR	561.646	703.891	564.982	708.895	3	2	y13	56	19	4	28.55
TARS	FLGDIEVWDQAEK	775.38	1004.468	779.387	1012.483	2	1	y8	141	33	44	37.94
TARSL2	WLWSEVER	552.777	805.384	557.781	815.392	2	1	y6	86	25	8	34.92
VARS	EAFLQEVWK	575.301	689.362	579.308	697.376	2	1	y5	76	23	14	36.48
WARS	GIFGFTDSDC*IGK	708.827	623.774	712.834	627.781	2	2	y11	111	27	14	35.06
YARS	EYTLDVYR	529.761	552.278	534.765	562.286	2	1	y4	66	21	12	24.49

^a^ Light peptides represent endogenous peptides.

^b^ Heavy peptides represent SIS peptides.

^c^ Declustering potential.

^d^ Collisional energy.

^e^ Collisional cell exit potential.

^f^. Retention time.

*: Carbamidomethylated cysteine

**Fig 3:**
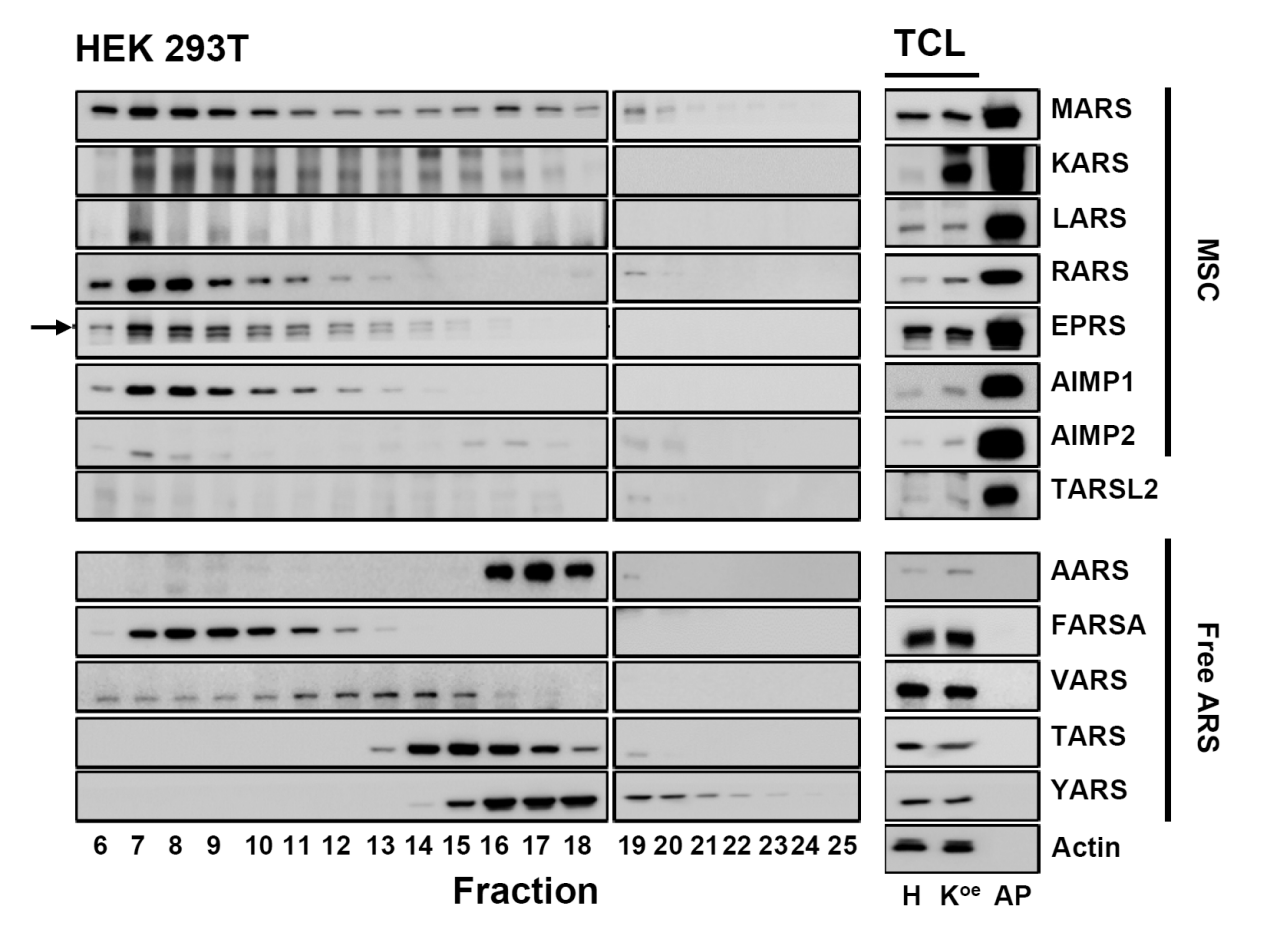
Expression level of endogenous ARS proteins in HEK 293T fractions by western blot analysis. All fractions (5 μL, respectively) were analyzed by western blot with selected ARS antibodies (left panel). Total cell lysate (10 μg) of HEK 293T (H) and KARS^oe^ (K^oe^), and 5μg of affinity-purified proteins of KARS^oe^ cells (AP) were used as a positive control (right panel). Actin was used as a loading control for total cell lysate. MSC, multi-tRNA synthetase complex; TCL, total cell lysate. Black arrow indicates EPRS.

The heat maps shown in Fig 2 present the amount of 25 ARS proteins (20 synthetases, 3 auxiliary proteins, and 2 variants) in HEK 293T, KARS^oe^ and KARS^oe^-AP fractions (Fig 2B, 2C and 2D). In case of HEK 293T and KARS^oe^, 8 ARS proteins and 3 auxiliary proteins, which are known as MSC components, were detected in the earlier fractions (from 6 to 10). The elution volume of these fractions was close to the void volume of the SEC column employed in the study. Free ARSs (such as TARS, NARS, etc.) were detected in the later fractions (from 13 to 23), the exact position depending on their molecular weight. The overall MRM profile of ARSs was quite similar between HEK 293T and KARS^oe^ except for those of KARS and leucyl-tRNA synthetase (LARS) (Fig 2B and 2C). We calculated correlation coefficient between HEK 293T and KARS^oe^. For this, we took the MRM signals of 10 MSC components except KARS in the fractions 6 to 10 (S2 and S3 Tables). The values we found were >0.91 for all the five fractions indicating that KARS overexpression did not affect the overall stoichiometry of MSC severely. When KARS was overexpressed, a large portion of KARS was eluted around fraction 16, presumably due to exclusion of extra KARS from MSC formation. Some portions of LARS were also eluted around fraction 16 in case of KARS^oe^. The mechanism for this phenomenon is yet unknown. Even though KARS and LARS participate in MSC formation in executing their canonical function, various non-canonical functions have also been investigated for which the ARSs need not form MSC. It is hypothesized that the large amount of extra KARS may be related with non-canonical function of LARS by hitherto unknown mechanism. To confirm the MRM-MS data and to detect the expression level of selected MSC components (MARS, KARS, LARS, RARS, EPRS, AIMP1, AIMP2 and TARSL2) and free ARSs (AARS, FARSA, VARS, TARS and YARS), western blot was executed. The western blot results were highly consistent with the MRM-MS profiles in HEK 293T and KARS^oe^ (Fig 3, left panel and S3 Fig). This result suggests that MRM-MS analysis based on SEC is effective for detection and quantification of intact protein complex in native condition.

In our previous paper, we reported TARSL2 as a potential member of MSC and found AIMP2-DX2, an alternatively spliced variant of AIMP2, in affinity-purified KARS of HEK 293T cells [9]. In order to further confirm the interaction of the two proteins with MSC, we monitored their unique surrogate peptides as well as those of canonical ARSs by MRM-MS in all experiments. Consistent with our previous report, TARSL2 and AIMP2-DX2 were detected in earlier fractions where MSC components were found (Fig 2B). The two proteins were still found in the same SEC fractions after affinity purification suggesting that they are part of MSC. Threonyl-tRNA synthetase (TARS) was detected in later fractions unlike TARSL2 (Figs 2 and 3). Though TARSL2 has 74% of homology with TARS in amino acid sequence, it possesses a unique N-terminal extension compared to TARS. We tentatively attribute the interaction of TARSL2 with MSC to the N-terminal extension. Since the expression level of TARSL2 is quite lower than TARS and other MSC components (Fig 3), MSC seems to be a dynamic complex in which a small portion of the complex has one more extra component like TARSL2. Our observation that AIMP2-DX2 as well as AIMP2 was co-purified with MSC does not guarantee that both isoforms form a single MSC. AIMP2-DX2 was reportedly upregulated in lung cancer progression [25]. Moreover, the variant competed with AIMP2 and inhibited tumor suppressive interaction of AIMP2 with TRAF2 [26]. It is reasonable to infer from the previous finding that AIMP2-DX2 may also compete with AIMP2 in MSC formation. However, since two molecules of AIMP2 participate in MSC [9, 27], our current data does not notify the exact stoichiometry of AIMP2-DX2-containing MSC. An outstanding merit of MRM-MS compared to immunoassay is its high selectivity. To the best of our knowledge, there is no commercially available antibody that can discriminate AIMP2-DX2 from its canonical isoform. Since the two isoforms differ slightly in their molecular weights, researchers have used antibodies specific to both isoforms after separating the proteins by SDS-PAGE [25, 26]. In contrast, MRM-MS enabled simultaneous detection of both AIMP2 and AIMP2-DX2 in a single sample without further separation.

**Fig 4:**
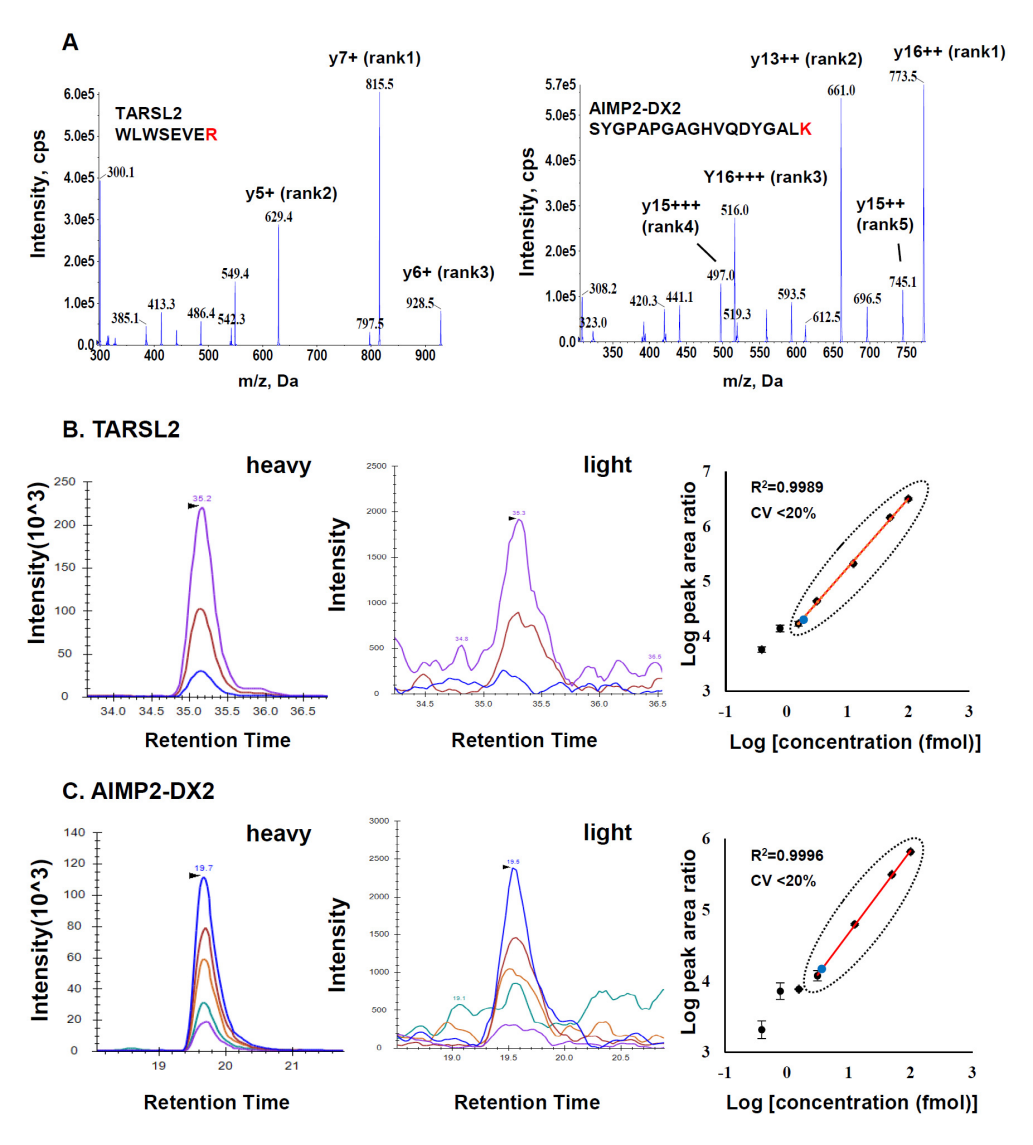
MS/MS spectra, extracted ion chromatograms, and calibration curves of TARSL2 and AIMP2-DX2 surrogate peptides. **(A)** MS/MS spectra of SIS peptides for TARSL2 (left) and AIMP2-DX2 (right) after optimization of MRM parameters (Heavy isotope-labeled amino acid is colored red in the peptide sequence). Three fragment y-ions of TARSL 2 (y7+, y5+, y6+) and five fragment y-ions of AIMP2-DX2 (y16++, y13++, y16+++, y15+++, y15++) used for peak assignment and quantitation are annotated within the spectra. **(B and C)** MRM chromatograms and calibration curves for quantifying TARSL2 (B) and AIMP2-DX2 (C). XIC of the TARSL2 (three y-ions) and AIMP2-DX2 (five y-ions) for SIS peptides (left panel) and for endogenous peptides in seventh SEC fraction of HEK 293T (middle panel) are presented. A dilution series of the SIS peptides for TARSL2 (y7+) and AIMP2-DX2 (y16++) (right panel) were analyzed in triplicated MRM runs and the resultant MRM peak areas are plotted as a function of peptide amount (right panel). The straight line within the plots represents linear response range with R^2^ ≥0.99 and CV ≤ 20% in which the lowest value corresponds to LOQ. Blue circles represent endogenous peptides in seventh fraction.

TARSL2 and AIMP2-DX2 existed in lower amount than other canonical ARS proteins (S1 Fig and S2 and S3 Tables). Thus, we determined the limit of quantification (LOQ) for the two proteins based on a calibration curve obtained by using chemically synthesized SIS peptides (WLWSEVER for TARSL2 and SYGPAPGAGHVQDYGALK for AIMP2-DX2; Fig 4A). Firstly, MRM parameters for the SIS peptides were optimized. The same order of magnitude in fragment ion intensities were observed between SIS heavy peptides and endogenous light peptides (TARSL2: y7+, y5+ and y6+; AIMP2-DX2: y16++, y13++, y16+++, y15+++ and y15++), suggesting that the signal we monitored in HEK 293T was real (Fig 4B and 4C, left and middle panel). In the next step, SIS peptides were quantified in the range of 0.39–100 fmol and the quantified MRM signal was plotted against the amount of SIS peptide. We obtained linear responses from 1.56 to 100 fmol for WLWSEVER, and from 3.13 to 100 fmol for SYGPAPGAGHVQDYGALK, with CV ≤ 20% and R^2^ ≥ 0.99 from triplicated experiments (Fig 4B and 4C, right panel). Therefore, the LOQ values for TARSL2 and AIMP2-DX2 were determined to be 1.56 and 3.13 fmol, respectively. Conversely, the calibration curve was used to determine the amount of endogenous proteins. The amount of TARSL2, and AIMP2-DX2 was 1.75 fmol, and 4.07 fmol in 2 μL of fraction 7, respectively (Fig 4B and 4C, right panel). The MRM response curves for other ARS proteins including KARS were also determined by using a dilution series of their corresponding SIS peptides (S4 and S5 Figs). Our result implies that it is possible to detect and quantify such low abundance proteins as minor isoform or variant protein by MRM-MS.

Detection and quantification of isoforms or variant proteins is sometimes a significant challenge for immunological assay because antibody may not discriminate similar homologues and sequence variants arising from mutation. In the current study, MRM signal from SYGPAPGAGHVQDYGALK is specific to DX2 variant of AIMP2. By measuring multiple transitions and quantifying as low as ~4 fmol, we were able to discriminate the variant from wild-type canonical form. In other studies published recently, 11 isoform family of S100 protein in cancer cell and 3 isoforms of TGF beta were accurately detected by MRM assay [28, 29]. Therefore, it is worth mentioning here that MRM-MS is effective in quantifying multiple forms of proteins sharing similar sequences without using antibodies.

Three ARSs (VARS, FARSA and FARSB) were detected in earlier fractions than expected by their molecular weights (Figs 2 and 3). Valyl-tRNA synthetase (VARS) forms a high molecular weight complex with the four subunits of elongation factor (EF)-1H in mammalian cells [30–32]. It has been reported that VARS/EF-1H complex is made up two copies each of VARS and heavy form of EF-1H and retains a total mass of about 600 kDa. Phenylalanyl-tRNA synthetase (FARS) is known to be the most complex enzymes of the ARSs with the tetrameric subunit. The enzyme is a heterotetrameric (αβ)_2_ enzyme, consisting of 508 amino acids in the α subunit (FARSA) and 589 amino acids in the β subunit (FARSB). The multimeric subunit composition of cytoplasmic FARS is conserved from prokaryotes and eukaryotes [33, 34]. Since VARS and FARS complexes have high molecular weight in native condition, their components were detected in the fractions where MSC appeared. However, we observed a slightly altered SEC elution profile of these ARSs when KARS was overexpressed. We propose that this difference is not due to the interaction of VARS and FARSs with extra KARS since these ARSs disappeared after affinity purification of MSC as will be discussed below.

To reduce identification of false interactions, we purified MSC from KARS^oe^ cell lysate by streptavidin affinity purification. Affinity-based precipitation using SBP-streptavidin binding is known to be strong and very specific for identifying protein interactions [35]. As can be seen in the heat map of KARS^oe^-AP, two ARSs forming large complexes (VARS and FARS subunits) disappeared after affinity purification (Fig 2D, left panel). This indicates that the large complexes involving VARS or FARSA and FARSB are different from MSC. Other non-MSC ARSs were scarcely detected after affinity purification. Affinity purification recovered more than 10% of MSC components, if the recovery of bait protein (KARS in this study) was set to 100%. Unlike MSC-forming ARSs, less than 0.8% was recovered for such non-MSC ARSs (Fig 2D, right panel). Even such tiny amount eluted in the later SEC fractions, hence excluding the possibility of their temporal binding to MSC components. This phenomenon can be regarded to non-specific binding during bead-based affinity purification. For characterization of protein interactions, various affinity matrices, such as magnetic and agarose beads, are commonly used in AP-MS technique. In addition to bona fide interaction, however, identified proteins may include contaminant proteins, including those that bind nonspecifically to the bead matrices [9, 36, 37]. Unlike non-MSC ARSs mentioned above, TARSL2 and AIMP2-DX2 were recovered in similar amounts as compared to MSC ARSs, and they eluted in the SEC fractions where MSC appeared in all experiments (Fig 2B–2D). The result further supports our previous proposal that AIMP2-DX2 and TARSL2 interact with MSC.

In this study, we used SIS peptide to normalize the MRM signals of a single ARS throughout all SEC fractions. However, we were unable to determine the stoichiometry of MSC components in a single fraction. Variable digestion efficiency between proteins as well as within a single protein seems to be a major factor for the limitation. It is accepted that different peptides have variable digestion efficiency in MRM study, and this in turn will affect the final quantification [38]. Use of isotope-labelled proteins instead of isotope-labeled peptides would guarantee identical digestion efficiency between target proteins and the standards and thus improve the quantitation accuracy. Despite the above-mentioned limitation, our strategy of using protein-based SEC followed by LC-MRM-MS is highly efficient and specific for analyzing protein complexes or protein-protein interactions, especially, in case of differentiating isoforms and variants.

## Conclusion

In the present study, we combined SEC with MRM-MS proteomic analysis to detect all ARS proteins including MSC components and variants in a human cell line. Although we were unable to determine the stoichiometry of MSC components, the method is highly valuable due to the detection of proteins without using antibodies. By non-denaturing SEC, we separated MSC components from free ARS proteins, depending on their molecular weight. This chromatographic technique helped make the precise MRM-MS analysis for detection of complex-forming proteins. Moreover, low abundance variants, TARSL2 and AIMP2-DX2, were accurately measured and their participation in MSC formation was also confirmed. Antibody-based assay is sometimes very hard to differentiate protein isoforms or variants showing difference in a short a.a stretch. It is hypothesized that by exploiting high specificity of mass spectrometry-based assay, our strategy of SEC-MRM-MS will be helpful to decipher the composition of intact protein complex and to understand the behavior of minor isoforms or variant proteins.

## Supporting Information

S1 Fig Extracted ion chromatograms of 25 ARS proteins in HEK 293T. Extracted ion chromatogram (XIC) of endogenous (red) and the corresponding SIS (blue) peptides for 25 ARSs are represented.
(TIF)

S2 Fig Size-exclusion chromatograms of ARS proteins measured by SEC-MRM-MS. Three biological replicates for each of three different samples were analyzed. The title of each chromatogram is given by [protein name].[surrogate peptide].
(PDF)

S3 Fig Expression level of endogenous ARS proteins in KARS^oe^ fractions. All fractions (5 μl, respectively) were analyzed by western blot with selected specific ARS antibodies. MSC, multi-tRNA synthetase complex. Black arrowhead indicates EPRS.
(TIF)

S4 Fig MS/MS spectra, extracted ion chromatograms, and calibration curves of KARS. **(A)** A representative MS/MS spectrum of SIS peptide for KARS **(B and C)** XIC of KARS (three y-ions) for SIS peptides (B) and for endogenous peptides in seventh SEC fraction of HEK 293T (C) are presented. **(D)** Calibration curve of KARS. Blue circle represents endogenous peptide in seventh fraction.
(TIF)

S5 Fig Calibration curves of 22 ARS surrogate peptides. A dilution series of the SIS peptides for 22 ARS were analyzed in triplicated MRM runs and the resultant MRM peak areas are plotted as a function of peptide amount. The straight line within the plots represents linear response range with R^2^ ≥ 0.98 and CV ≤ 20% in which the lowest value corresponds to LOQ.
(TIF)

S1 Table Experimentally optimized MRM parameters of 38 surrogate peptides. (XLSX)

S2 Table The amount of endogenous ARS proteins in all fractions of HEK 293T. (XLSX)

S3 Table The amount of endogenous ARS proteins in all fractions of KARS^oe^. (XLSX)

S4 Table The amount of endogenous ARS proteins in all fractions of KARS^oe^-AP. (XLSX)

## Abbreviations

AAA: amino acid analysis
AIMP: aminoacyl tRNA synthetase complex-interaction multifunctional protein
AP-MS: affinity purification mass spectrometry
ARS: aminoacyl-tRNA synthetase
CE: collisional energy
CXP: collisional cell exit potential
DP: declustering potential
MRM: multiple reaction monitoring
MSC: multi-tRNA synthetase complex
SBP: streptavidin binding peptide
SEC: size exclusion chromatography
SIS: stable isotope standard
TARSL2: threonyl-tRNA synthetase-like 2
XIC: extracted ion chromatography
