# Supplementary figures and images for: Evaluation of Multi-tRNA Synthetase Complex by Multiple Reaction Monitoring Mass Spectrometry Coupled with Size Exclusion Chromatography
Source: PLoS One. 2015 Nov 6;10(11):e0142253. doi: 10.1371/journal.pone.0142253 (PMC4636271; doi:10.1371/journal.pone.0142253)

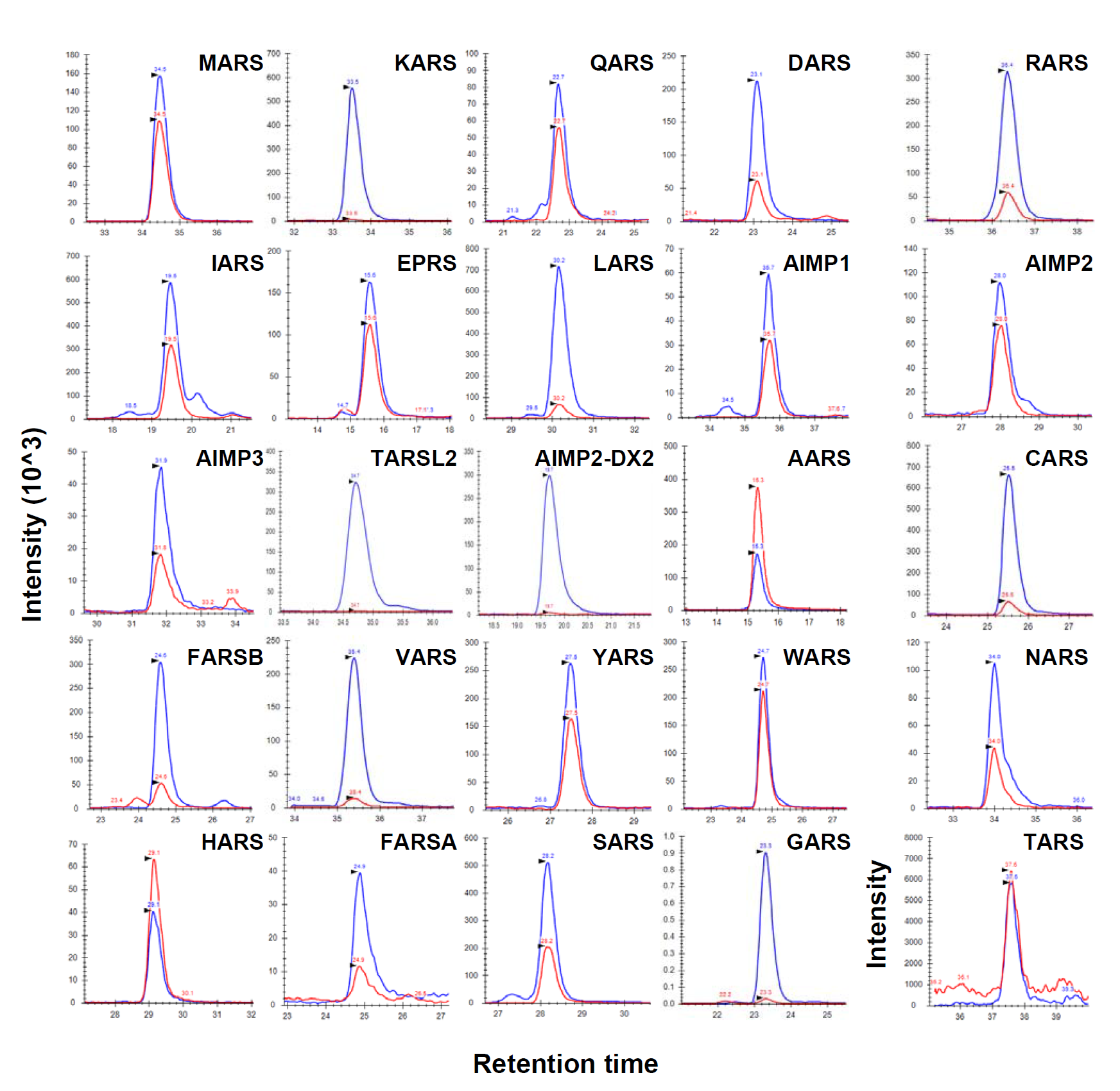

Supplement: S1 Fig — Extracted ion chromatogram (XIC) of endogenous (red) and the corresponding SIS (blue) peptides for 25 ARSs are represented. (TIF) [file pone.0142253.s001.tif]

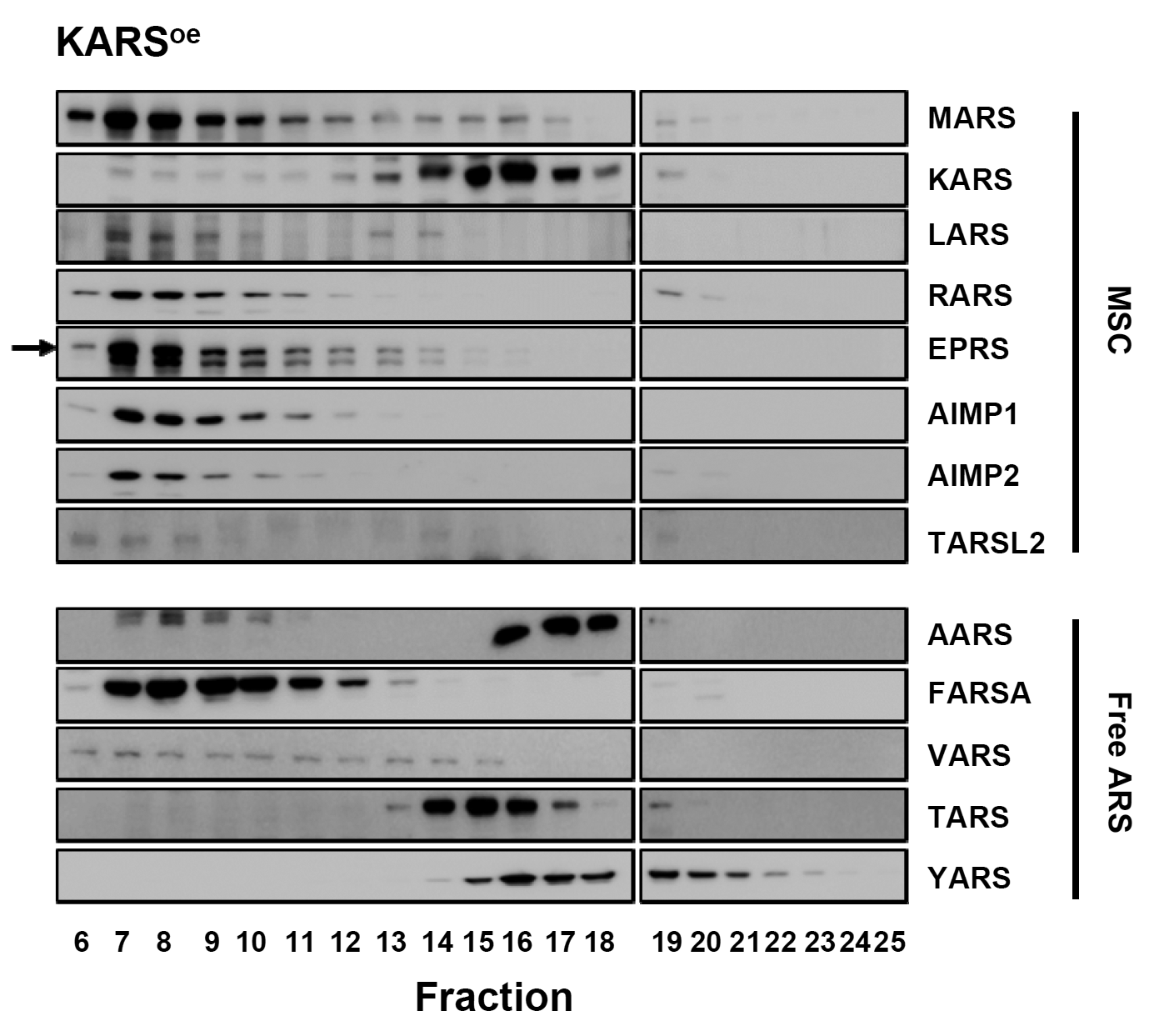

Supplement: S3 Fig — All fractions (5 μl, respectively) were analyzed by western blot with selected specific ARS antibodies. MSC, multi-tRNA synthetase complex. Black arrowhead indicates EPRS. (TIF) [file pone.0142253.s003.tif]

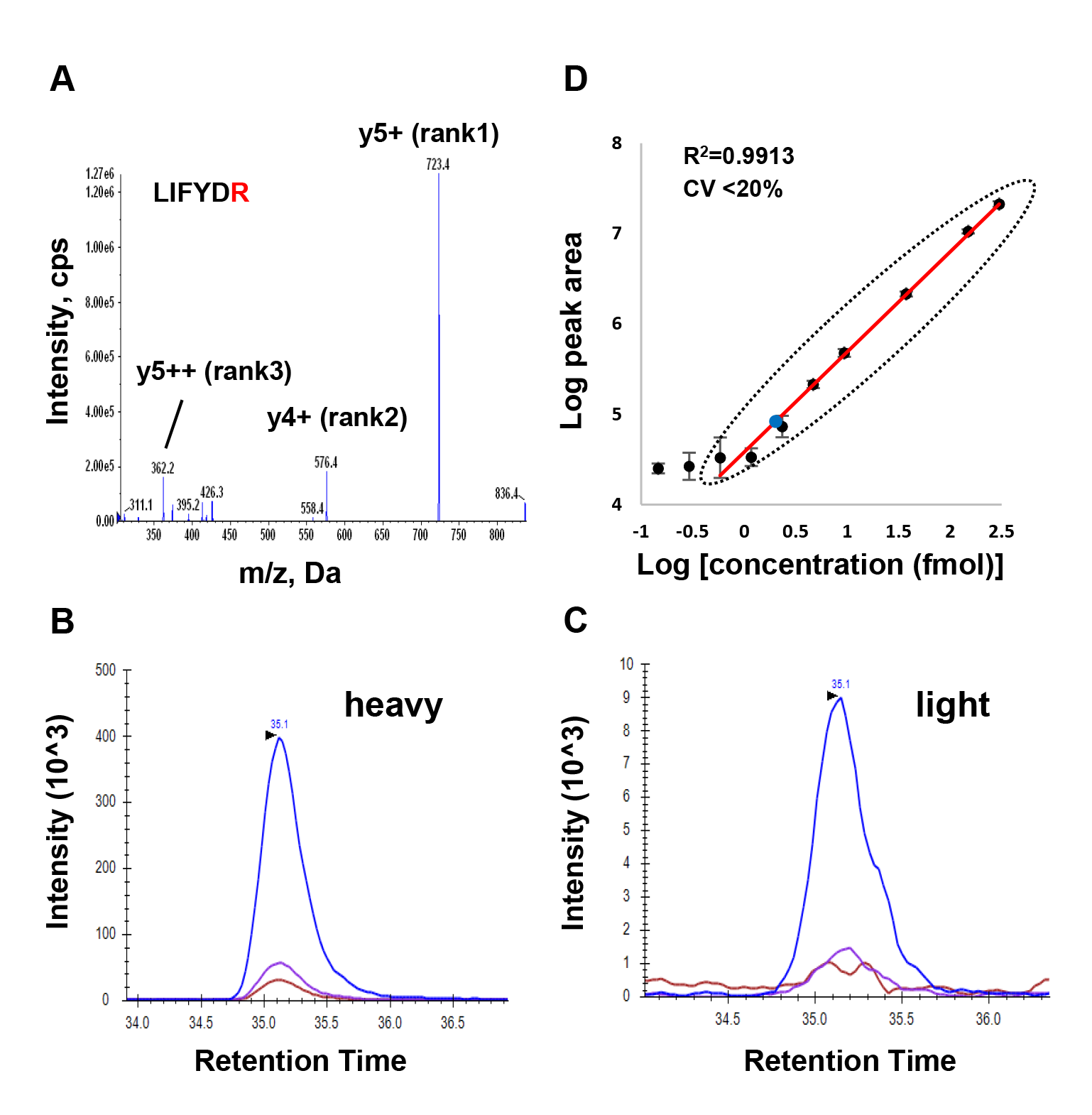

Supplement: S4 Fig — (A) A representative MS/MS spectrum of SIS peptide for KARS (B and C) XIC of KARS (three y-ions) for SIS peptides (B) and for endogenous peptides in seventh SEC fraction of HEK 293T (C) are presented. (D) Calibration curve of KARS. Blue circle represents endogenous peptide in seventh fraction. (TIF) [file pone.0142253.s004.tif]

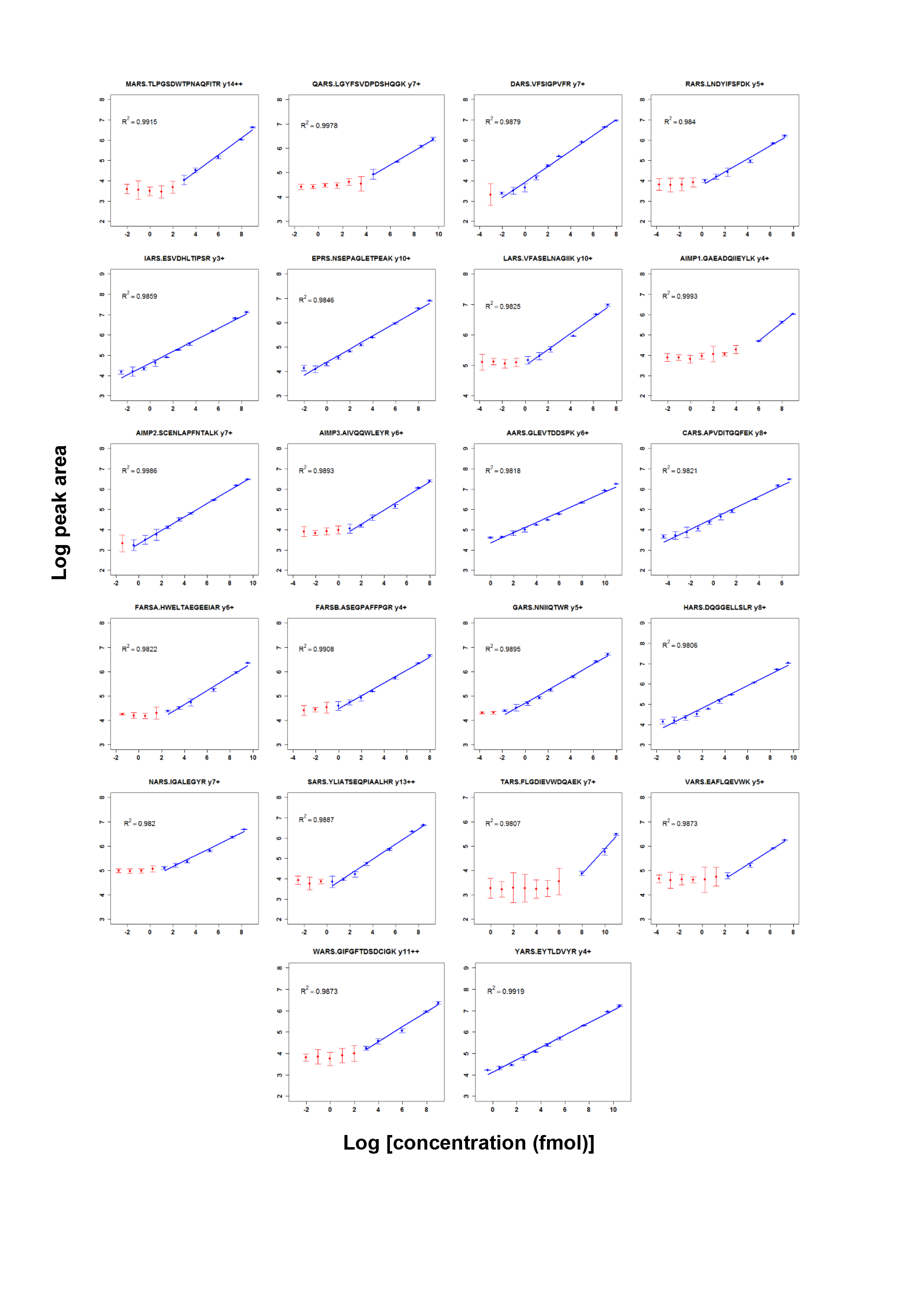

Supplement: S5 Fig — A dilution series of the SIS peptides for 22 ARS were analyzed in triplicated MRM runs and the resultant MRM peak areas are plotted as a function of peptide amount. The straight line within the plots represents linear response range with R2 ≥ 0.98 and CV ≤ 20% in which the lowest value corresponds to LOQ. (TIF) [file pone.0142253.s005.tif]
