# Supplementary material for: Evaluation of Multi-tRNA Synthetase Complex by Multiple Reaction Monitoring Mass Spectrometry Coupled with Size Exclusion Chromatography
Source: PLoS One. 2015 Nov 6;10(11):e0142253. doi: 10.1371/journal.pone.0142253 (PMC4636271; doi:10.1371/journal.pone.0142253)
Supplement: S2 Fig — Three biological replicates for each of three different samples were analyzed. The title of each chromatogram is given by [protein name].[surrogate peptide]. (PDF) [file pone.0142253.s002.pdf]

H: HEK 293T
K: KARS^oe
A: KARS^oe-AP

Experiment 1
Experiment 2
Experiment 3

PCC
: Pearson correlation coefficient

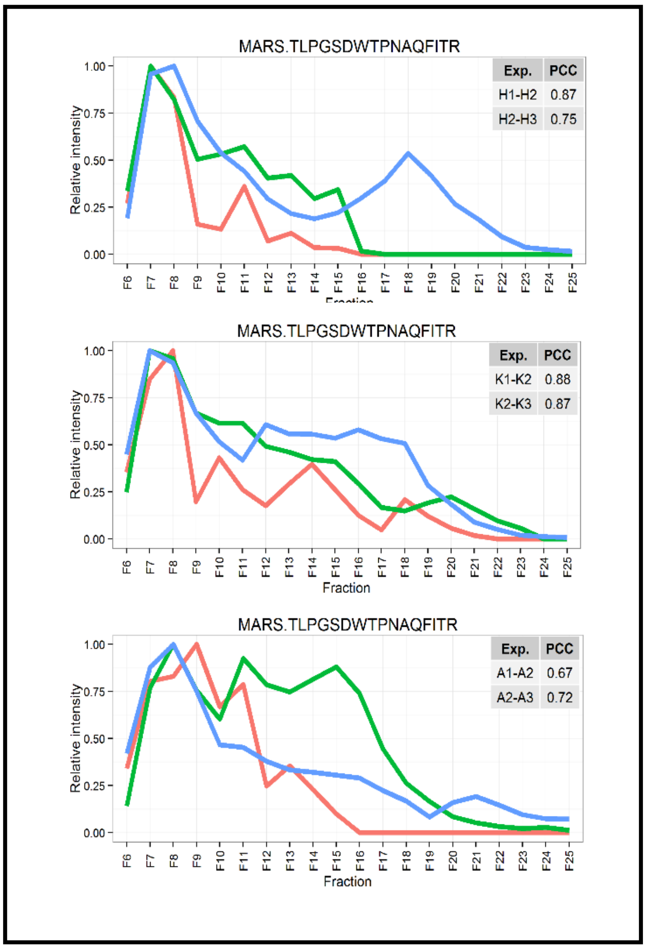

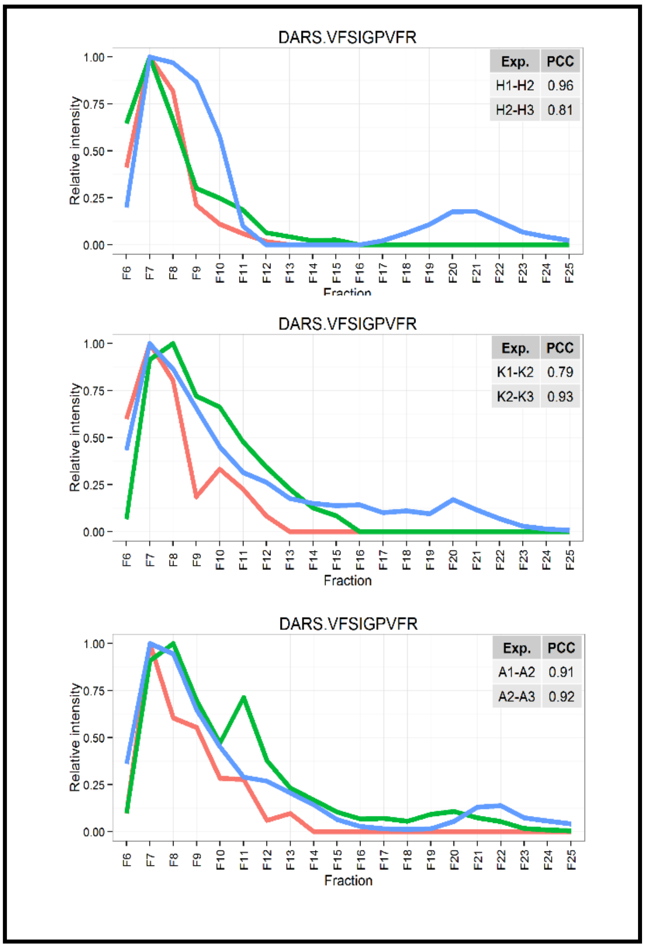

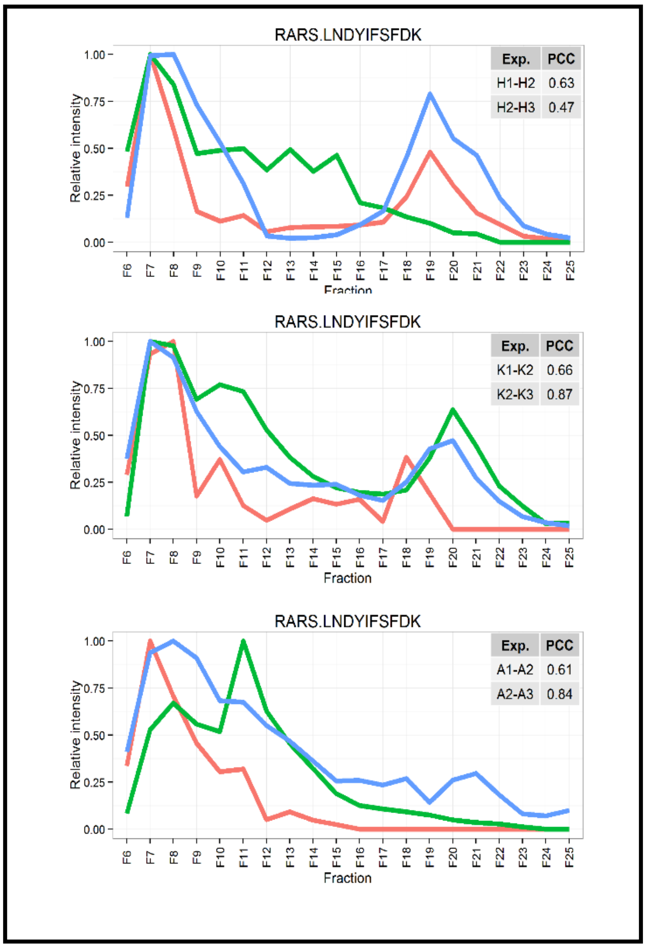

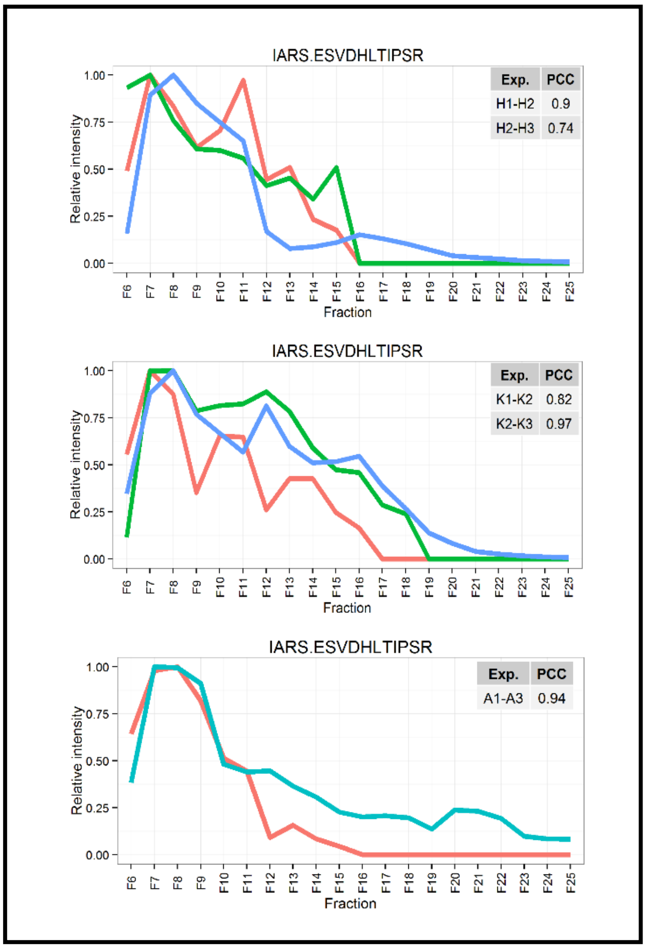
IARS.ESVDHLTIPSR
Exp. PCC
H1-H2 0.9
H2-H3 0.74
IARS.ESVDHLTIPSR
Exp. PCC
K1-K2 0.82
K2-K3 0.97
IARS.ESVDHLTIPSR
Exp. PCC
A1-A3 0.94
Relative intensity
Fraction
F6 F7 F8 F9 F10 F11 F12 F13 F14 F15 F16 F17 F18 F19 F20 F21 F22 F23 F24 F25
0.00 0.25 0.50 0.75 1.00

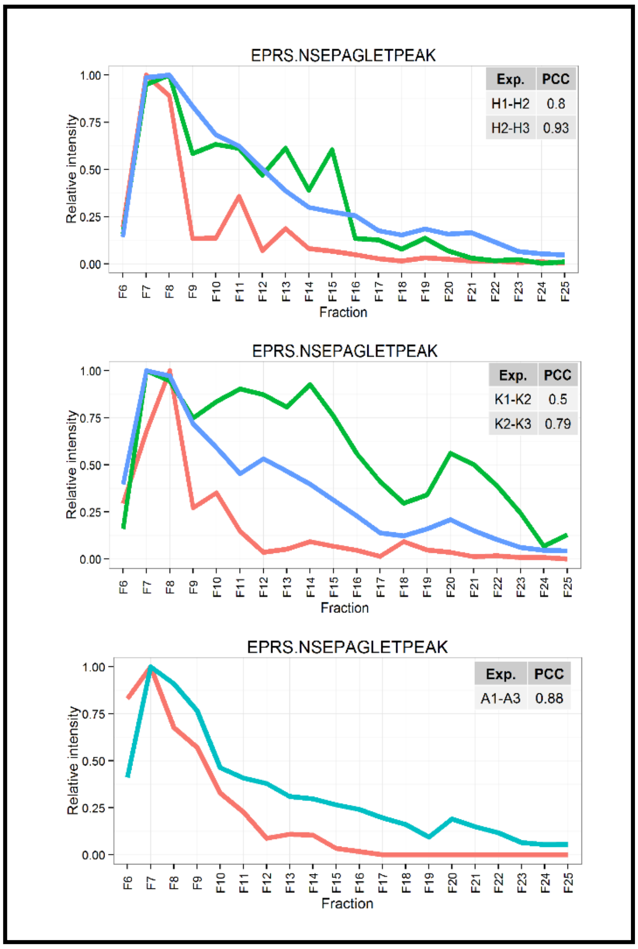
EPRS.NSEPAGLETPEAK
Exp. PCC
H1-H2 0.8
H2-H3 0.93
EPRS.NSEPAGLETPEAK
Exp. PCC
K1-K2 0.5
K2-K3 0.79
EPRS.NSEPAGLETPEAK
Exp. PCC
A1-A3 0.88
Relative intensity
Fraction
F6 F7 F8 F9 F10 F11 F12 F13 F14 F15 F16 F17 F18 F19 F20 F21 F22 F23 F24 F25
0.00 0.25 0.50 0.75 1.00

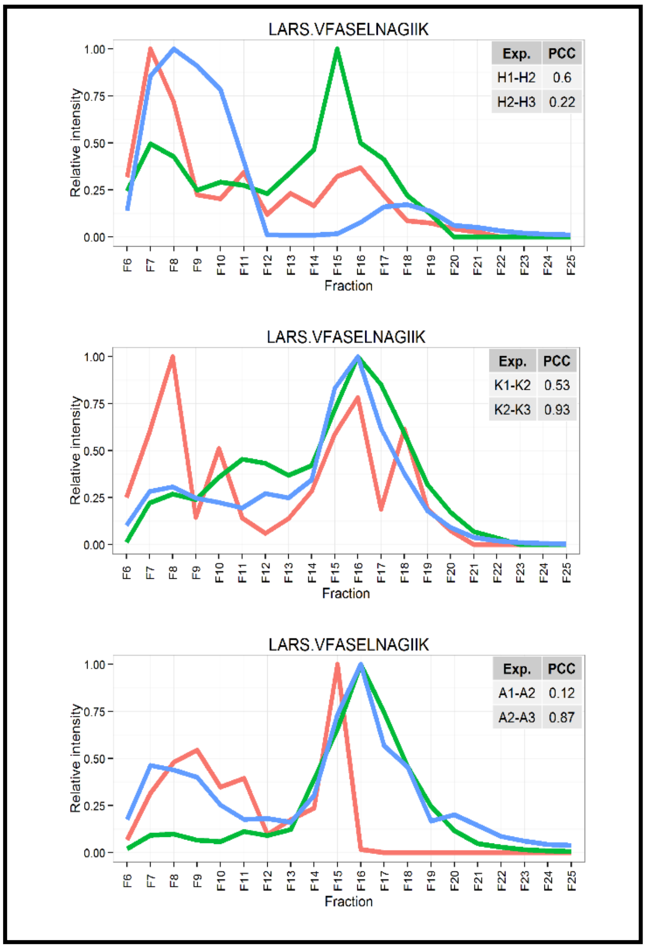
LARS.VFASELNAGIIK
Exp. PCC
H1-H2 0.6
H2-H3 0.22
LARS.VFASELNAGIIK
Exp. PCC
K1-K2 0.53
K2-K3 0.93
LARS.VFASELNAGIIK
Exp. PCC
A1-A2 0.12
A2-A3 0.87
Relative intensity
Fraction
F6 F7 F8 F9 F10 F11 F12 F13 F14 F15 F16 F17 F18 F19 F20 F21 F22 F23 F24 F25
0.00 0.25 0.50 0.75 1.00

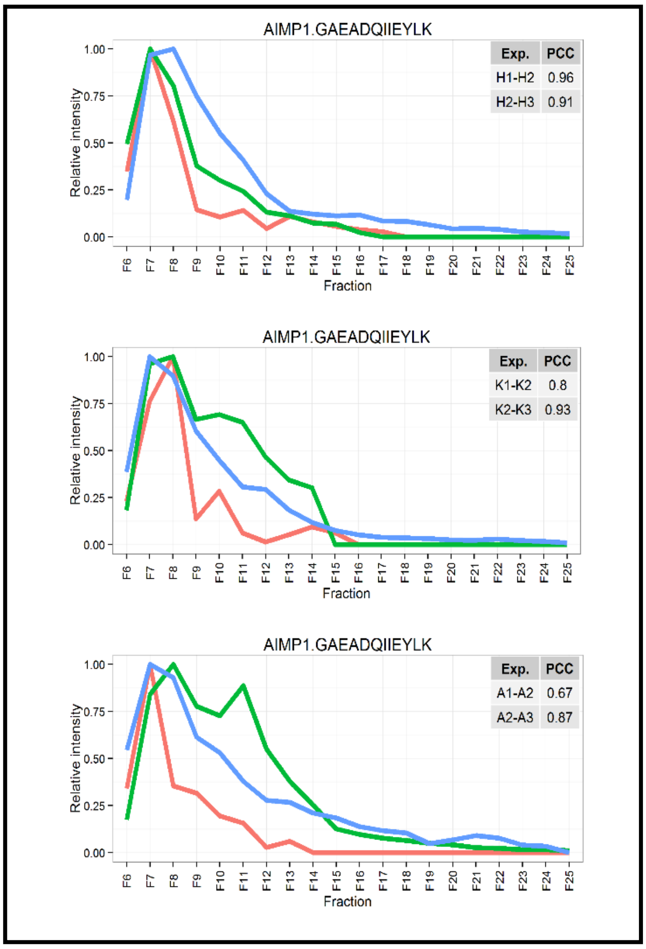
AIMP1.GAEADQIIEYLK
Exp. PCC
H1-H2 0.96
H2-H3 0.91
AIMP1.GAEADQIIEYLK
Exp. PCC
K1-K2 0.8
K2-K3 0.93
AIMP1.GAEADQIIEYLK
Exp. PCC
A1-A2 0.67
A2-A3 0.87
Relative intensity
Fraction
F6 F7 F8 F9 F10 F11 F12 F13 F14 F15 F16 F17 F18 F19 F20 F21 F22 F23 F24 F25
0.00 0.25 0.50 0.75 1.00

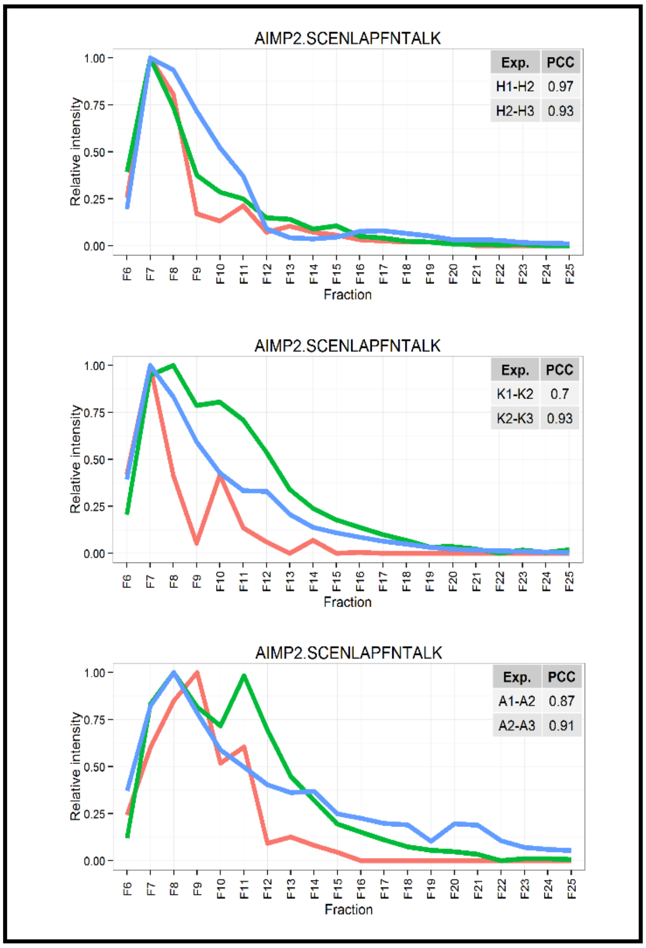
AIMP2.SCENLAPFNTALK
Exp. PCC
H1-H2 0.97
H2-H3 0.93
AIMP2.SCENLAPFNTALK
Exp. PCC
K1-K2 0.7
K2-K3 0.93
AIMP2.SCENLAPFNTALK
Exp. PCC
A1-A2 0.87
A2-A3 0.91
Relative intensity
0.00 0.25 0.50 0.75 1.00
F6 F7 F8 F9 F10 F11 F12 F13 F14 F15 F16 F17 F18 F19 F20 F21 F22 F23 F24 F25
Fraction

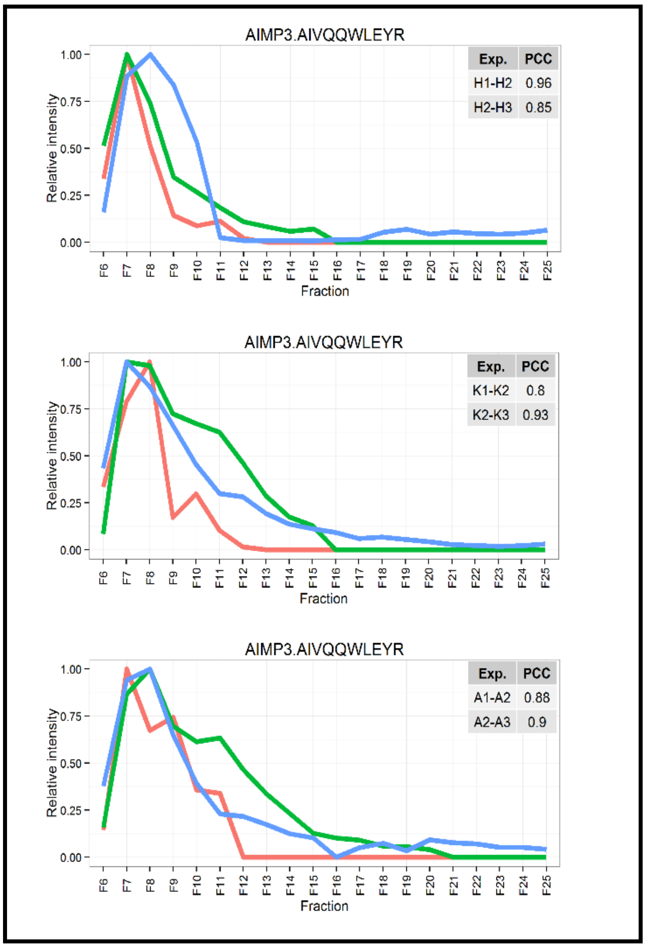
AIMP3.AIVQQWLEYR
Exp. PCC
H1-H2 0.96
H2-H3 0.85
AIMP3.AIVQQWLEYR
Exp. PCC
K1-K2 0.8
K2-K3 0.93
AIMP3.AIVQQWLEYR
Exp. PCC
A1-A2 0.88
A2-A3 0.9
Relative intensity
0.00 0.25 0.50 0.75 1.00
F6 F7 F8 F9 F10 F11 F12 F13 F14 F15 F16 F17 F18 F19 F20 F21 F22 F23 F24 F25
Fraction

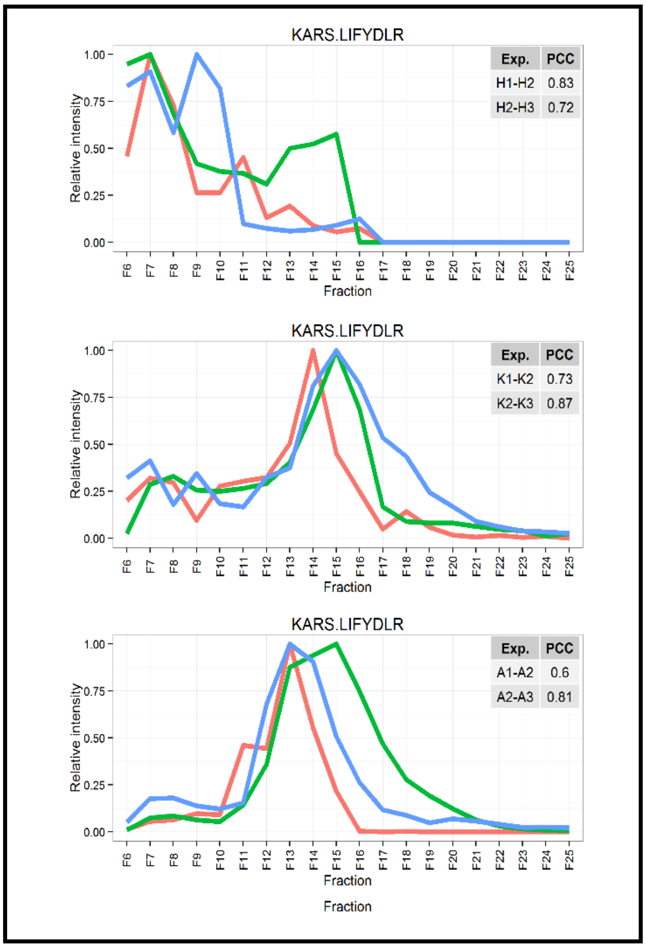
KARS.LIFYDLR
Exp. PCC
H1-H2 0.83
H2-H3 0.72
KARS.LIFYDLR
Exp. PCC
K1-K2 0.73
K2-K3 0.87
KARS.LIFYDLR
Exp. PCC
A1-A2 0.6
A2-A3 0.81
Relative intensity
0.00 0.25 0.50 0.75 1.00
F6 F7 F8 F9 F10 F11 F12 F13 F14 F15 F16 F17 F18 F19 F20 F21 F22 F23 F24 F25
Fraction
Fraction

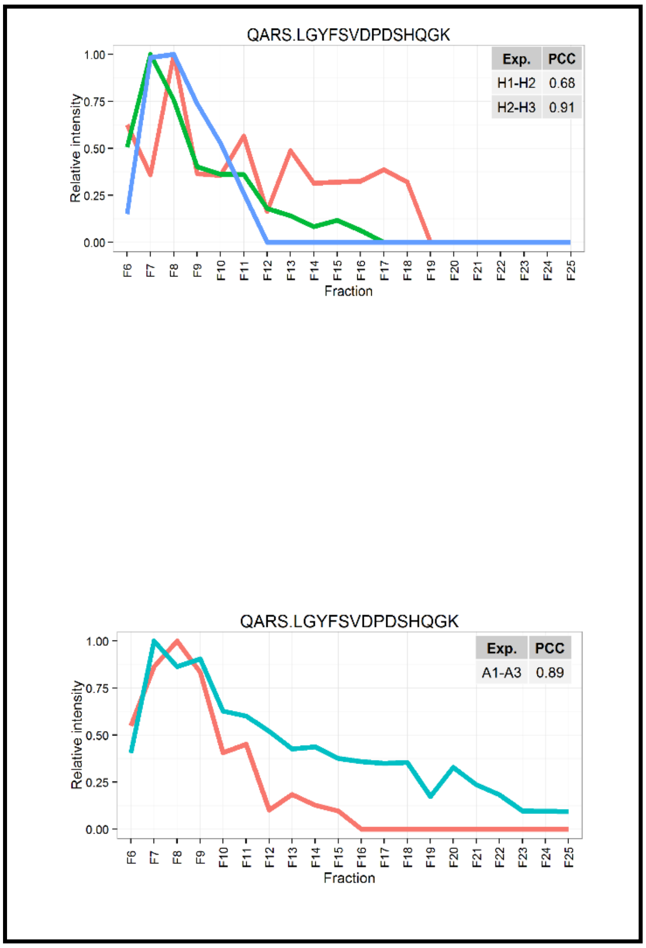
QARS.LGYFSVDPDSHQGK
Exp. PCC
H1-H2 0.68
H2-H3 0.91
QARS.LGYFSVDPDSHQGK
Exp. PCC
A1-A3 0.89
Relative intensity
0.00 0.25 0.50 0.75 1.00
F6 F7 F8 F9 F10 F11 F12 F13 F14 F15 F16 F17 F18 F19 F20 F21 F22 F23 F24 F25
Fraction

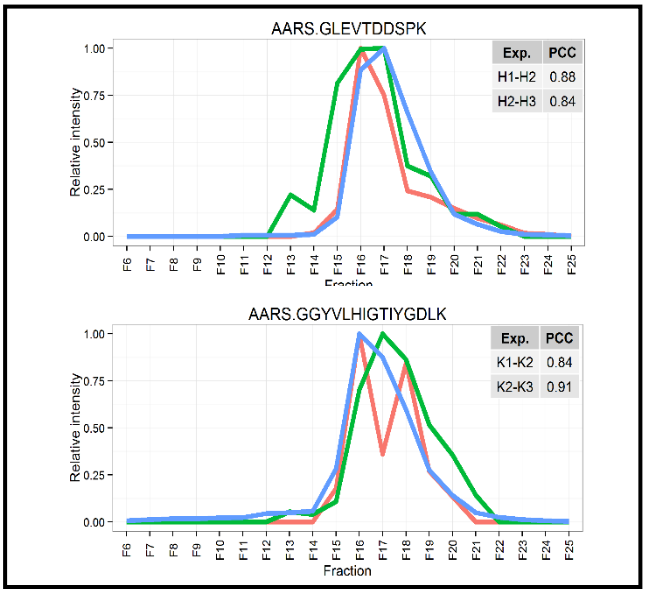
AARS.GLEVTDDSPK
Exp. PCC
H1-H2 0.88
H2-H3 0.84
AARS.GGYVLHIGTIYGDLK
Exp. PCC
K1-K2 0.84
K2-K3 0.91
Relative intensity
Fraction

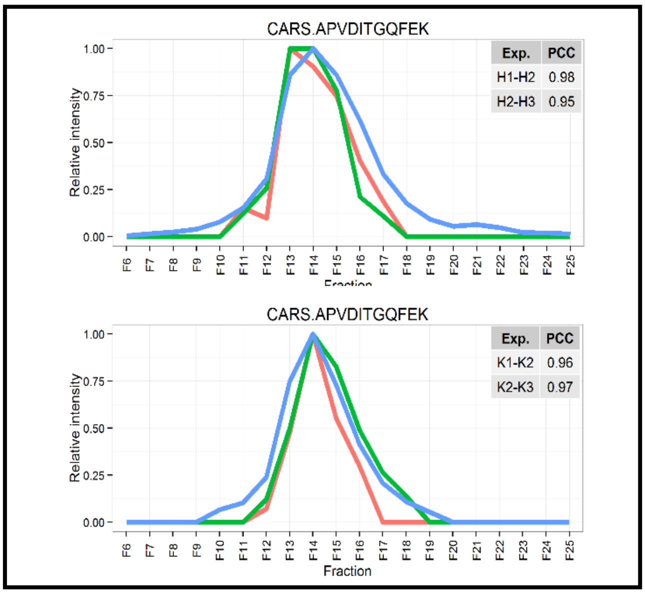
CARS.APVDITGQFEK
Exp. PCC
H1-H2 0.98
H2-H3 0.95
CARS.APVDITGQFEK
Exp. PCC
K1-K2 0.96
K2-K3 0.97
Relative intensity
Fraction

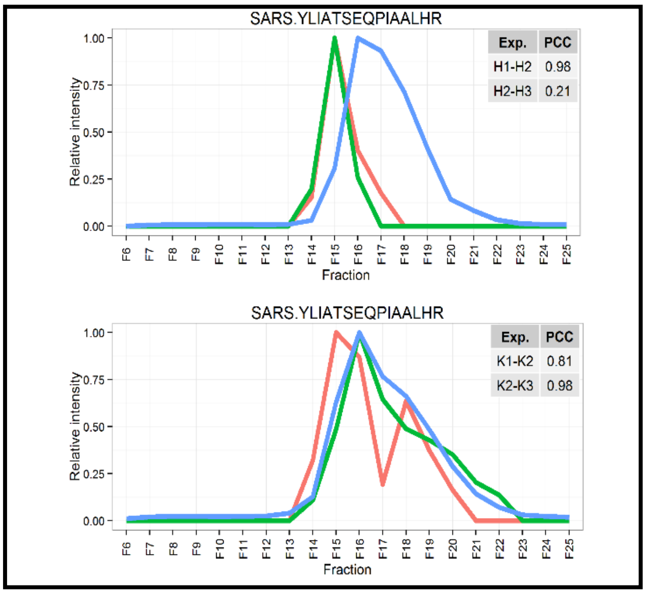
SARS.YLIATSEQPIAALHR
Exp. PCC
H1-H2 0.98
H2-H3 0.21
SARS.YLIATSEQPIAALHR
Exp. PCC
K1-K2 0.81
K2-K3 0.98
Relative intensity
Fraction

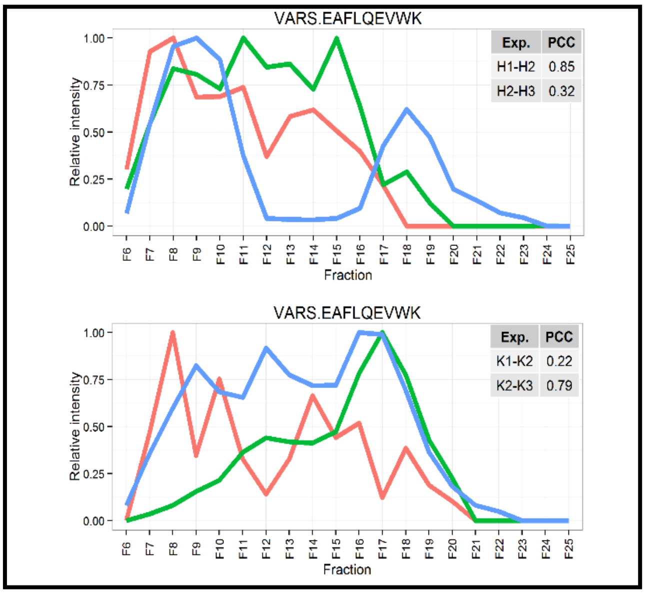
VARS.EAFLQEVWK
Exp. PCC
H1-H2 0.85
H2-H3 0.32
VARS.EAFLQEVWK
Exp. PCC
K1-K2 0.22
K2-K3 0.79
Relative intensity
Fraction

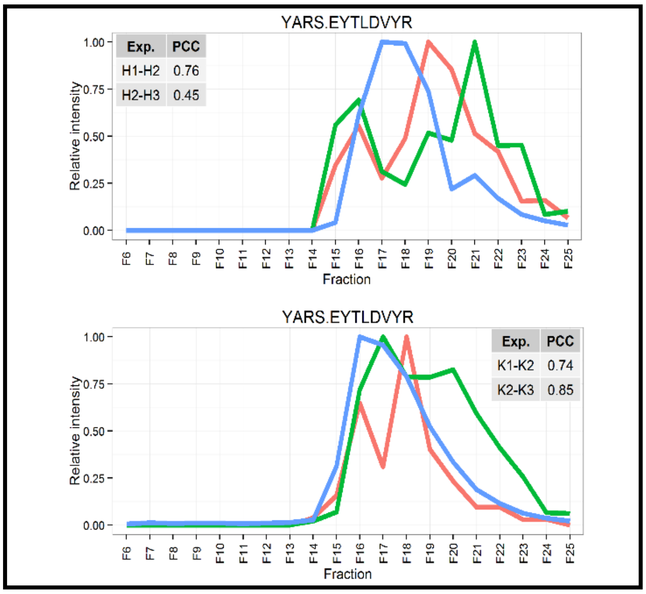
YARS.EYTLDVYR
Exp. PCC
H1-H2 0.76
H2-H3 0.45
YARS.EYTLDVYR
Exp. PCC
K1-K2 0.74
K2-K3 0.85
Relative intensity
Fraction

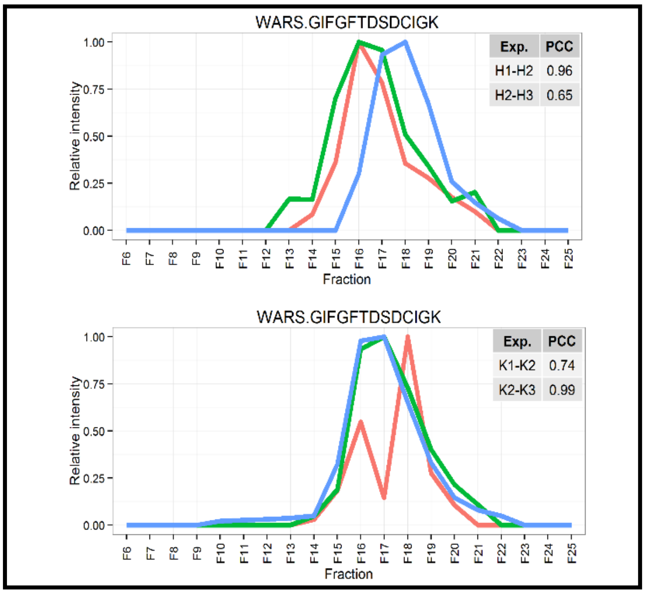
WARS.GIFGFTDSDCIGK
Exp. PCC
H1-H2 0.96
H2-H3 0.65
WARS.GIFGFTDSDCIGK
Exp. PCC
K1-K2 0.74
K2-K3 0.99
Relative intensity
Fraction

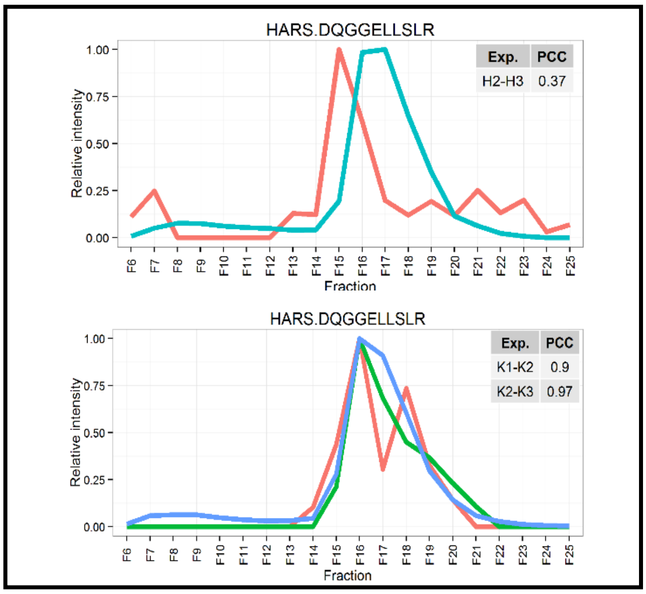
HARS.DQGGELLSLR
Exp. PCC
H2-H3 0.37
HARS.DQGGELLSLR
Exp. PCC
K1-K2 0.9
K2-K3 0.97
Relative intensity
Fraction

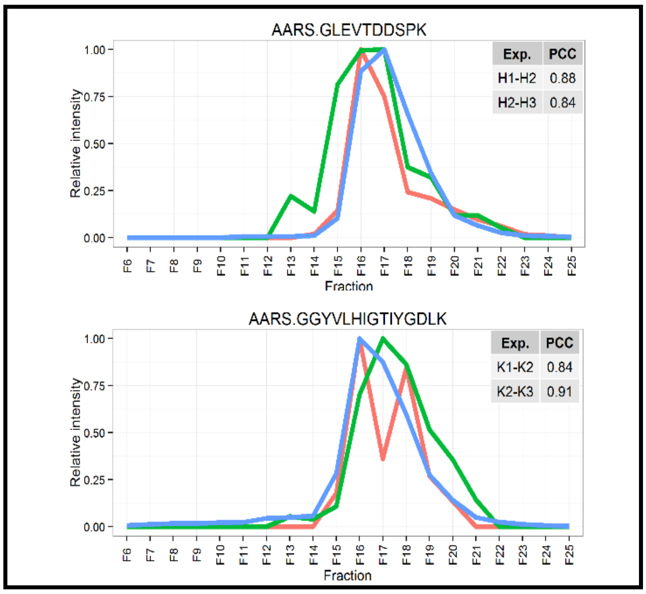
AARS.GLEVTDDSPK
Exp. PCC
H1-H2 0.88
H2-H3 0.84
AARS.GGYVLHIGTIYGDLK
Exp. PCC
K1-K2 0.84
K2-K3 0.91
Relative intensity
Fraction

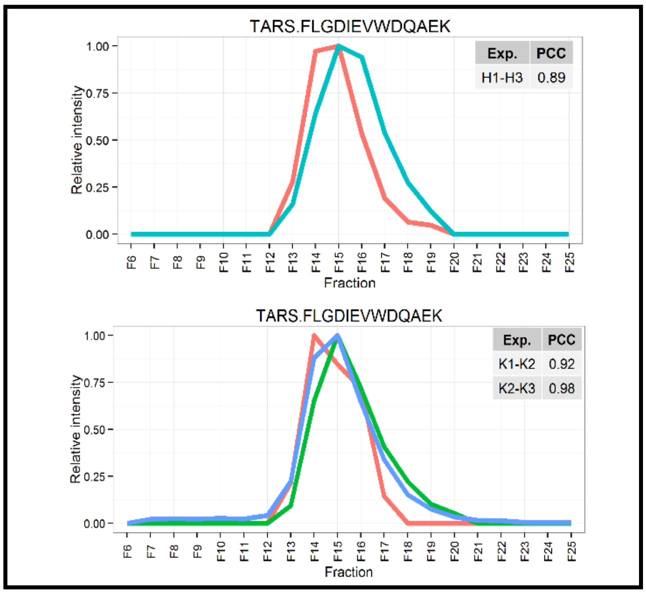
TARS.FLGDIEVWDQAEK
Exp. PCC
H1-H3 0.89
TARS.FLGDIEVWDQAEK
Exp. PCC
K1-K2 0.92
K2-K3 0.98
Relative intensity
Fraction

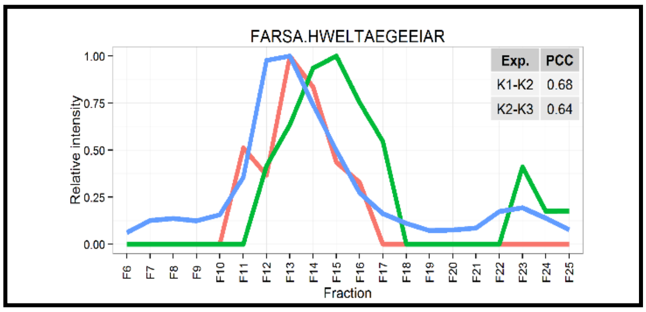
FARSA.HWELTAEGEEIAR
Exp. PCC
K1-K2 0.68
K2-K3 0.64
Relative intensity
Fraction

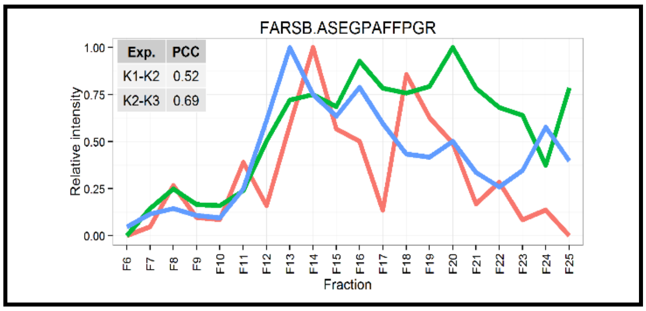
FARSB.ASEGPAFFPGR
Exp. PCC
K1-K2 0.52
K2-K3 0.69
Relative intensity
Fraction

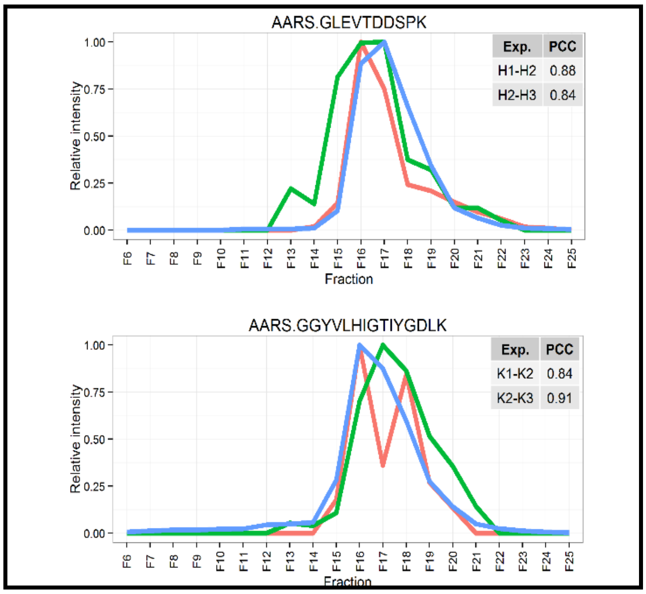
AARS.GLEVTDDSPK
Exp. PCC
H1-H2 0.88
H2-H3 0.84
AARS.GGYVLHIGTIYGDLK
Exp. PCC
K1-K2 0.84
K2-K3 0.91
Relative intensity
Fraction

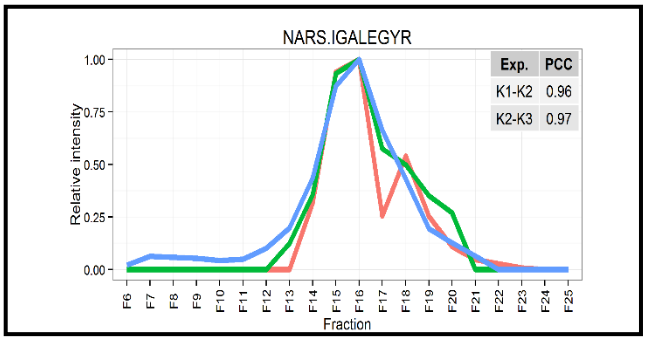
NARS.IGALEGYR
Exp. PCC
K1-K2 0.96
K2-K3 0.97
Relative intensity
Fraction
